# Melifoliox B, a novel phloroglucin derivative isolated from *Melicope barbigera* (Rutaceae) and synthesis of new oxidation products from melifoliones A and B

**DOI:** 10.3762/bjoc.22.39

**Published:** 2026-03-24

**Authors:** Horst Weber, Kim-Thao Tran-Cong, Bernhard Mayer, Guido J Reiss, Iryna S Konovalova, Marc S Appelhans, Kenneth R Wood, Claus M Passreiter

**Affiliations:** 1 Institute of Pharmaceutical and Medicinal Chemistry, Heinrich-Heine-University Duesseldorf, 40225 Duesseldorf, Germany; 2 Institute of Pharmaceutical Biology and Biotechnology, Heinrich-Heine-University Duesseldorf, 40225 Duesseldorf, Germany; 3 Institute of Organic Chemistry and Macromolecular Chemistry, Heinrich-Heine-University Duesseldorf, 40225, Duesseldorf, Germany; 4 Institute of Inorganic Chemistry and Structural Chemistry, Heinrich-Heine-University Duesseldorf, 40225 Duesseldorf, Germany; 5 Integrative Taxonomy of Plants, Friedrich-Schiller-University Jena, 07743 Jena, Germany; 6 Senckenberg Institute for Plant Form and Function (SIP) Jena, 07743 Jena, Germany; 7 National Tropical Botanical Garden, 3530 Papalina Road, Kalaheo, HI 96741, USA

**Keywords:** *Melicope barbigera*, Melifoliones A and B, new heterocyclic ring systems, new natural compounds, *para*-quinols, phenol oxidation

## Abstract

In addition to new acetophenones and 2*H*-chromenes, the dichlormethane extract from leaves of *Melicope barbigera* A. Gray (Rutaceae) afforded a mixture of the isomeric melifoliones A (**1**) and B (**2**) as well as an oxidation product of **2**, whose structure was elucidated as the *para*-quinol **4**. For an independent synthesis of **4** and its possible isomer **3**, the required compounds **1** and **2** were synthesized as a mixture of the isomers starting from chromene **5**, briefly heated in a closed microwave apparatus with catalytic amounts of acetic acid. Forced heating of **5** in acetic acid or use of stronger acids lead to the benzoxocin derivatives **6** and **7**. Oxidation of melifoliones **1** and **2** under a great variety of oxidants and conditions failed to give **3** and **4**. Iodine-containing oxidants yielded the products **8**, **9**, and **10**. Combined oxidation with hydrogen peroxide and ferricyanide in alkaline solution resulted in an unexpected contraction of the acetyl phenol to a furanone ring, forming the derivatives **11** and **12**, whose structures were confirmed by X-ray analysis. A hypothetical mechanism for the oxidative ring contraction is proposed. **11** and **12** are the first representatives of new heterocyclic ring systems that have not previously been described in the literature.

## Introduction

The genus *Melicope* is a member of the Rutaceae (Citrus family) and contains more than 200 species distributed in the Malagasy, Indo-Himalayan, South-East Asian and Pacific regions. More than 50 endemic species of *Melicope* are found on the Hawaiian Islands and belong to the section Pelea. *Melicope* species are proven to produce many interesting secondary metabolites including poly-methoxylated flavonoids, furocoumarins, acetophenones and quinolone alkaloids. Moreover, several *Melicope* species are used in traditional and modern medicine. Some of the constituents possess antibacterial, antidiabetic, cytotoxic and antiproliferative activities in human cancer lines [1].

Five years ago, we described new acetophenones and chromenes isolated from a dichloromethane extract of leaves of *Melicope barbigera* A. Gray, a species endemic to the island of Kaua’i, Hawaiian Islands [2–3]. In addition, small amounts of two tetracyclic citrans were identified as a 30:70%-mixture of isomeric melifoliones A (**1**), and melifolione B (**2**) [1], both formerly found in *Melicope latifolia* (syn. *Euodia latifolia* [4].

**Figure 1:**
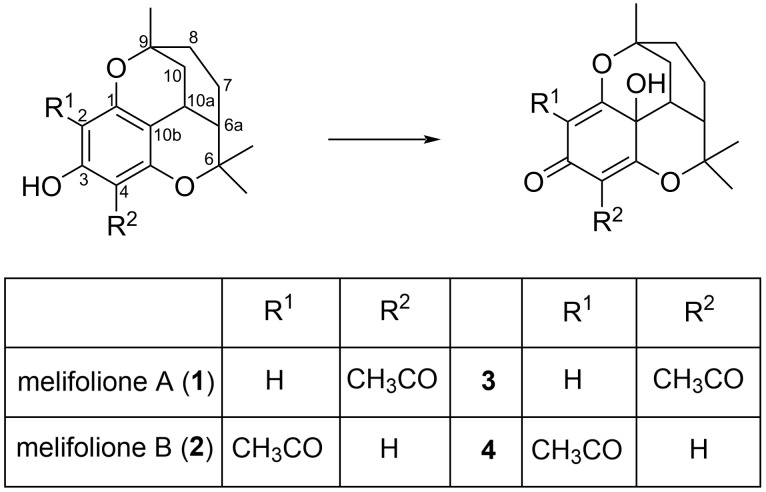
Structures of isomeric melifoliones and corresponding oxidation products.

We now report on the identification and structure elucidation of a new natural compound **4** in the dichloromethane extract of leaves of *Melicope barbigera*, which was characterized as an oxidation product of melifolione B (**2**) by means of high resolution electrospray ionization mass spectrometry (HRESIMS) and NMR spectra. However, the isomeric compound **3** could not be detected in the extract (Figure 1).

Since **4** could be an artefact, built by oxidation of **2** during working up of the extract, and to finally confirm the structure, the isomeric melifoliones should be synthesized first, to get enough quantity for oxidation to the corresponding quinols in a second experiment. Using the method of Wang and Lee [5], **1** could be prepared regio-specifically in good yield by heating phloroacetophenone and citral in DMF under catalysis of ethylenediammonium diacetate (EDDA). However, synthesis of **2** was difficult. Despite numerous attempts, we only were able to get mixtures of both melifoliones with minor amounts of **2**. Since all attempts to separate both isomers on a preparative scale failed, we were only able to record NMR spectra from a very small quantity of **2**, received via fractional crystallization. However, the oxidation procedures were performed with mixtures of both isomers with the aim to get the quinols **3** and **4**.

## Results and Discussion

### Structure of compound **4**

Compound **4** was isolated from a dichloromethane extract of the leaves of *M. barbigera* as a colorless and amorphous substance. The molecular formula was determined as C_18_H_22_O_5_ by high resolution electrospray ionization mass spectrometry (HRESIMS), thus containing one more oxygen atom than the melifoliones. The UV spectrum showed a maximum at 250 nm, typical for an α,β-unsaturated carbonyl structure.

The ^1^H NMR spectrum of **4** showed signals for 22 protons (Table 1) with a certain similarity to the melifoliones, but with an exchangeable signal found at δ 2.88 ppm, typical for a tertiary carbinol, instead of a phenolic OH-group at about δ 13 ppm, found in the spectra of the melifoliones. All 18 signals detected in the ^13^C NMR spectrum of **4** (Table 2) could be assigned by careful analysis of the 2D-NMR spectra (HSQC and HMBC). The position of the acetyl group was confirmed by a cross peak between C-2 and the protons of the acetyl-methyl group in the HMBC spectrum and by interactions between the acetyl-methyl group and the methyl group at C-9 as well as between H-4 and one of the geminal methyl groups at position 6 in the NOESY-spectrum. This allowed us to clarify the structure of **4** as an oxidation product of melifolione B (**2**). We suggest the following name: 2-acetyl-6a,7,8,9,10,10a-hexahydro-10b-hydroxy-6,6,9-trimethyl-1,9-epoxy-6*H*-dibenzo[*b*,*d*]pyran-3(10b*H*)-one and also propose the common name: melifoliox B for this new natural compound.

**Table 1:** ^1^H-NMR data of melifolione A (**1**), melifolione B (**2**) and compound **4** in CDCl_3_ (600 MHz, δ in ppm).

Position	**1**	**2**	**4**

H-2	6.04 (s)	–	–
H-4	–	6.01 (s)	5.77 (s)
H-6a	2.09 (ddd)^a^	2.02 (ddd)^a^	2.74 (t)
H_2_-7	0.86/1.31 (m)	0.87/1.30 (m)	1.69/1.79 (m)
H_2_-8	1.45/1.80 (m)	1.45/1.80 (m)	1.55/1.68 (m)
H-10_ax_	1.86 (dd)^b^	1.86 (dd)^b^	1.26 (m)
H-10_eq_	2.19 (ddd)^c^	2.19 (ddd)^c^	1.36 (m)
H-10a	2.73 (br. s)	2.83 (br. s)	2.34 (dt)
6-CH_3 ax_	1.13 (s)	1.07 (s)	1.46 (s)
6-CH_3 eq_	1.59 (s)	1.51 (s)	1.54 (s)
9-CH_3_	1.40 (s)	1.45 (s)	1.24 (s)
CH_3_CO	2.62 (s)	2.62 (s)	2.61 (s)
3-OH	13.3 (s)	13.6 (s)	–
10b-OH	–	–	2.88 (s)

*J* in Hz: ^a^11.7/5.3/2.7; ^b^13.3/1.6; ^c^13.3/4.5/3.5

**Table 2:** ^13^C NMR data of compounds **1**, **2** and **4** in CDCl_3_ (150 MHz, δ in ppm).

Position	**1**	**2**	**4**	HMBC correlations of **4**

C-1	162.8	158.9	195.2	C-1/H-10a
C-2	97.1	106.4	107.5	C-2/H-4; C-2/CH_3_-CO
C-3	164.2	165.3	190.5	–
C-4	107.4	98.5	106.3	–
C-4a	159.4	163.4	171.5	C-4a/H-10a
C-6	86.8	85.2	88.5	C-6/6-CH_3__ ax_; C-6/6-CH_3 eq_; C-6/H-6a
C-6a	46.4	46.1	35.8	C-6a/6-CH_3 ax_; C-6a/6-CH_3 eq_; C-6a/H-10a
C-7	22.0	22.1	24.7	C-7/H-6a
C-8	37.6	37.8	36.2	C-8/H-6a; C-8/9-CH_3_; C-8/H_2_-7
C-9	76.2	76.7	70.6	C-9/9-CH_3_; C-9/H_2_-7; C-9/H_2_-10
C-10	35.0	34.9	37.0	C-10/H-10a; C-10/9-CH_3_
C-10a	27.7	27.5	44.1	C-10a/H-6a; C-10a/H-7_eq_; C-10a/H_2_-10
C-10b	107.5	106.6	72.5	C-10b/H-4
6-CH_3 ax_	24.6	24.3	28.3	6-CH_3 ax_/H-6a; 6-CH_3 ax_/6-CH_3 eq_
6-CH_3 eq_	30.1	29.7	31.7	6-CH_3 eq_/H-6a; 6-CH_3 eq_/6-CH_3 ax_
9-CH_3_	28.8	29.0	26.5	9-CH_3_/H_2_-8; 9-CH_3_/H_2_-10
CH_3_-CO	32.2	32.6	28.2	–
CH_3_-CO	202.3	202.9	201.5	CH_3_-CO/CH_3_-CO

The stereochemistry of **4** is characterized by the three centers of asymmetry of the isomeric melifoliones at position 6a, 9, and 10a. Since the two hydrogenated pyran rings of **4** can only be linked cis, the two possible enantiomers of **4** have the following configuration: (6a*R*,9*S*,10a*S*,10b*R*) or (6a*S*,9*R*,10a*R*,10b*S*). Due to the finding, that the isolated compound showed no optical rotation, it exists as a racemate.

A stereo model of **4** provided important information for a better understanding of the NMR spectra. In contrast to the planar benzene ring of melifoliones, the para quinol ring in **4** adopts a flat but rigid boat conformation. However, the sp^3^-hybridized C-10b leads to greater flexibility of the whole molecule. Therefore, it is easier for the cyclohexane ring to deviate from a chair to a twisted boat conformation by rotating around the C-7/C-8-axis. As a result, the orientation of the geminal protons at these positions can change more easily between a pseudo-axial (ax) and a pseudo-equatorial (eq) position, and the difference in chemical shift values of these protons decreases compared to melifolione B (**2**). However, all protons in a pseudo-axial position show the expected high field shift compared to the pseudo-equatorial positions (Table 1).

The biggest change in chemical shift values of **4** compared to **2** is observed in the geminal protons at C-7 and C-10. While the protons at C-7 in **2** are strongly shielded by the aromatic ring current effect and therefore appear at the highest field, this effect is absent in **4**. The protons at C-10 of **4** come to resonance at the highest field. With the greater flexibility of the cyclohexane ring of **4**, the geminal methyl groups in the pyran ring can fold more easily to avoid the steric hindrance caused by the ethane bridge above the pyran rings. Consequently, these methyl protons resonate at similar field strength. The high field shift of H-10a in **4** is a result of the loss of its benzylic position (Table 1). The chemical shift values of C-6a and C-10a in the ^13^C NMR spectrum of **4** compared to **2** can be explained by the effect of the new OH-group at C-10b. The γ-position of C-6a leads to a high field shift (from 46.1 to 35.8 ppm) while C-10a shows the opposite effect (from 27.5 to 44.1 ppm), due to its β-position in regard to the OH-group at C-10b (Table 2).

### Synthesis of melifoliones

Melifolione A (**1**) was prepared according to the method of Wang and Lee [5] by heating phloroacetophenone and citral with ethylene-diammonium diacetate (EDDA) in DMF. However, reaction of the educts in pyridine at 60 °C led to chromene **5**, which is a strong phenol. It is therefore easily separable as a nearly pure compound from the complex reaction mixture by extraction of the ether solution with NaOH. According to the literature, **5** should be converted into melifolione B (**2**) under acidic conditions [6].

**Scheme 1:**
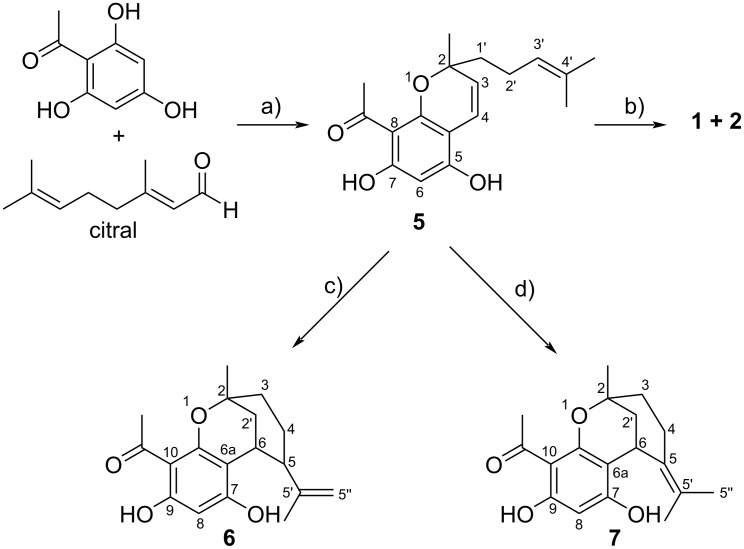
Synthesis of melifoliones and by-products: a) pyridine/60 °C/8 h/51%, b) microwave/140 °C/20 min/70%, c) acetic acid/80 °C/1 h/57%, d) *p*-toluenesulfonic acid/toluene/80 °C/1 h/68%.

Numerous attempts were made to cyclize **5** into melifolione B (**2**) with the following results (Scheme 1)

- Heating of **5** in DMF at 100 °C yielded melifolione A (**1**) with traces of melifolione B (**2**). When **5** was heated in acetic acid at 80 °C, only benzoxocin **6** could be isolated.
- Reaction of **5** with catalytic amounts of *p*-toluenesulfonic acid at 80 °C in toluene afforded benzoxocin (**7**) as the only product. On the other hand, melifolione A (**1**) was completely decomposed under these conditions.
- Irradiation of **5** in methanol with UV-light (300 nm) for 3 days at room temperature gave melifolione B (**2**) with traces of melifolione A (**1**), but the yield was less than 5% and therefore of no preparative interest.
- The best results to get melifolione B (**2**) were achieved by briefly heating **5** in a closed microwave apparatus without solvent at 130–140 °C after addition of catalytic amounts of acetic acid. By this procedure, the proportion of **2** in the mixture of melifoliones was increased to a maximum of about 10–15%.

Since it was not possible to distinguish or even to separate the isomeric melifoliones using chromatographic methods, their quantitative ratio after cyclization of **5** could only be determined using the ^1^H NMR spectra. After careful analysis of the 2D NMR spectra (HSQC, HMBC) all carbon signals of both isomers could be unambiguously assigned to the corresponding atoms (Table 2). This clearly shows that the data given in literature [4] must be corrected.

### Oxidation of melifoliones

**Figure 2:**
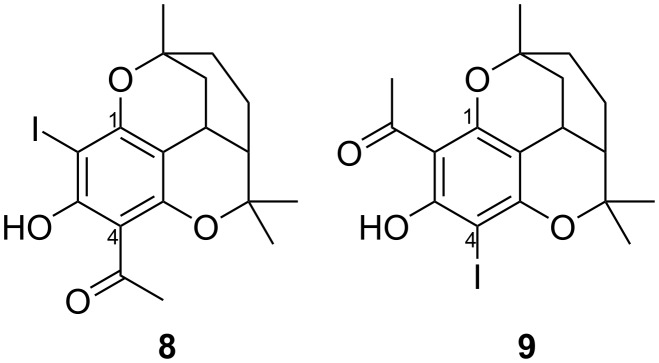
Structure of iodized melifoliones.

Mixtures of melifolione A (**1**) and melifolione B (**2**) (ratio ca. 10:1) were used for the following oxidation procedures:

- After oxidation with alkaline ferricyanide or using the Fenton reaction with iron(II) sulfate/hydrogen peroxide in acidic solution, only the unchanged melifoliones could be recovered in both cases.
- Oxidation with potassium dichromate in acidic solution or with potassium permanganate in acidic or alkaline solution led to complete decomposition of the melifoliones.
- A solution of the melifoliones in methanol with addition of methylene blue decomposed completely into undefined products within 24 hours when exposed to sunlight and atmospheric oxygen.
- Oxidation with potassium peroxodisulfate (Oxone^®^) according to a method by Carreño et al. [7] led to a complex mixture of compounds in which the quinols **3** and **4** could be excluded.
- While oxidation with alkaline hypochlorite solution gave a mixture of not identified chlorinated products, reaction with alkaline Lugol’s solution yielded the mono-iodized isomers of both melifolione A (**8**) and melifolione B (**9**), identified by their NMR spectra (Figure 2).
- Reaction with iodosobenzene diacetate – a typical reagent to form *para*-quinols from phenols [8] – yielded small amounts of a substance, that could be identified as iodized phenyl ether of melifolione A (**10**), based on its HRESIMS and ^1^H NMR spectrum. The quinols **3** or **4** could not be detected.

**Scheme 2:**
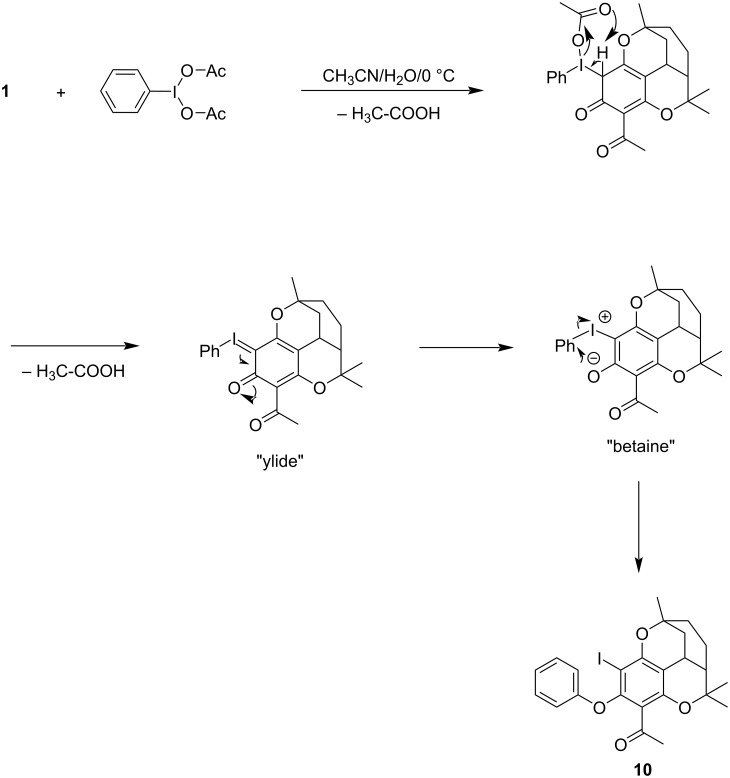
Reaction of melifolione A (**1**) with iodosobenzene diacetate.

Phenols with additional electronegative substituents (e.g., nitro or acetyl) and at least one *ortho*-positioned hydrogen atom initially react with iodosobenzene diacetate to form iodonium ylides or betaines, which, however, are unstable and react to form stable iodized phenolic ethers [9] (Scheme 2). In the ^1^H NMR spectrum of compound **10**, the signals for the phenolic OH and for the aromatic proton are missing compared to melifolione A (**1**). In contrast, the aromatic protons of the added phenyl ring appear as a typical coupling pattern in the expected range for phenyl ethers. The acetyl-methyl group is shifted by 0.3 ppm to a higher field and the methyl group in position 9 resonates at the same field strength as in the case of 2-iodomelifolione A (**8**).

Since all attempts to prepare the quinols **3** or **4** via oxidation of melifoliones had failed, the combined use of ferricyanide and hydrogen peroxide in alkaline solution was finally used. Under these conditions, superoxide and hydroxy radicals or even singlet oxygen can be formed [10–11]. After addition of an alkaline solution of ferricyanide to a hydrogen peroxide containing solution of the melifoliones (**1**:**2** ratio ca. 10:1) in acetonitrile, the color turns deep purple and later orange. After consumption of educts (TLC), extraction with ether yielded small amounts of a new substance as glistening crystals. The high-resolution mass spectrum gave a protonated mol peak at 279.1226 indicating the formula C_15_H_19_O_5_. Compared to melifoliones this means a loss of 3 C-atoms. Based on the NMR spectra (^1^H, 2D-COSY, ^13^C, HSQC, HMBC) the structure of the oxidation product was determined as the spirofuranone **11**.

The ^1^H NMR spectrum of **11** (Table 3), compared to melifoliones, is characterized by the loss of the signals for the phenolic OH- and the acetyl-methyl-group and it is complex in the range of 1.3 to 1.7 ppm. Only the evaluation of the HSQC spectrum revealed that the singlet for the equatorial 1-methyl group and the center of the multiplet for the axial H-5 lie exactly on the top of each other at 1.61 ppm. The same applies to the signals of both axial H-7 and H-8 at 1.57 ppm. The signal for H-8a appears as a nearly symmetrical quintet, resulting from the *trans* diaxial coupling with H-8_ax_ (about 10 Hz) and two gauche couplings with H-4a and H-8_eq_ (each about 5 Hz). In addition to the typical vicinal couplings, two ^4^*J*-long-range couplings can be recognized in the 2D-COSY spectrum of **11**. Both pairs of equatorial protons at C-5 and C-7 and at C-4a and C-8 are in a so-called W-arrangement, typical for a rigid chair conformation of the cyclohexane ring.

**Table 3:** NMR data of **11** in CDCl_3_ (^1^H = 600 MHz, ^13^C = 150 MHz, δ ppm).

Position	^1^H	^13^C	HMBC correlations

1	–	83.5	C-1/1-CH_3_ (2x), C-1/H_2_-8
3	–	165.2	C-3/H-4’ (^4^J), C-3/1-CH_3_ (2x ^4^J)
4 = 2’	–	80.4	C-4/H-4’, C-4/H-4a, C-4/H-5_ax_, C-4/H-8a
4a	3.12	33.4	C-4a/H-8a, C-4a/H-8_eq_
5	1.61^a^/1.92	35.0	C-5/6-CH_3_, C-5/H_2_-7
6	–	86.0	C-6/H-4a, C-6/H_2_-5, C-6/H_2_-7, C-6/H_2_-8, C-6/6-CH_3_
7	1.57^a^/2.10	34.9	C-7/6-CH_3_
8	1.57^a^/1.99	19.1	C-8/H-4a, C-8/H-7_ax_
8a	1.86	39.4	C-8a/H-5_eq_, C-8a/H-7_eq_, C-8a/1-CH_3_ (2x)
1-CH_3 ax_	1.42	26.7	1-CH_3 ax_/1-CH_3 eq_
1-CH_3 eq_	1.61^a^	27.6	1-CH_3 eq_/1-CH_3 ax_
6-CH_3_	1.40	28.4	6-CH_3_/H-5_ax_, 6-CH_3_/H-7_eq_
3’	–	179.4	C-3’/H-4’, C-3’/6-CH_3_ (^4^J)
4’	5.52	101.4	-
5’	–	172.2	C-5’/H-4’

^a^Signals are not separated and only detectable in the HSQC-spectrum.

The ^13^C NMR spectrum of **11** (Table 3) shows 15 signals that could be clearly assigned after evaluating the HSQC and HMBC spectra. In the HMBC spectrum, cross peaks occur for C-3 with H-4’ and with the protons of both geminal CH_3_ groups at C-1. Furthermore, a cross peak was found for the interaction of C-3’ with the protons of the 6-CH_3_ group. These observations suggest unusual (^4^*J*)-long-range C/H-couplings in the rigid spirocyclic 4-ring system.

**Scheme 3:**
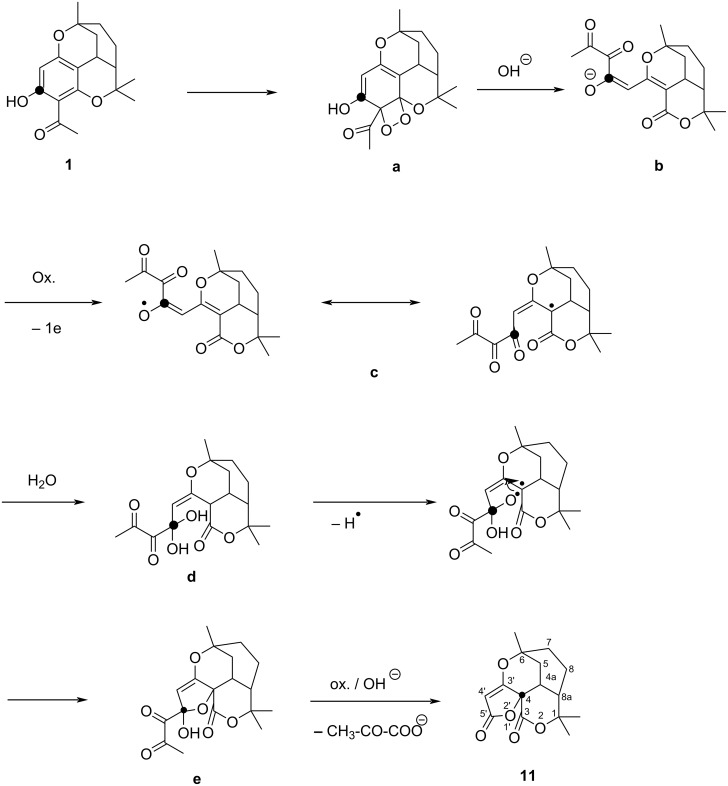
Hypothetical pathway for the oxidative ring contraction of melifolione A (**1**) with ferricyanide/hydrogen peroxide.

The formation of **11** can be suggested by the following hypothetical pathway (Scheme 3): In a first step, the combination of hydrogen peroxide with ferricyanide reacts with the phenol in a dioxygenase like reaction [12] to give a dioxetane **a**, followed by opening of the aromatic ring to form the oxonol anion **b** in alkaline solution, thus explaining the purple color. Oxidation of **b** leads to the delocalized radical **c**. Further oxidation of the hydrate **d** results in the spirofuran **e** [13]. After ferricyanide oxidation of **e,** the 3-C dicarbonyl residue splits off as pyruvate [14], leaving the spirofuranone **11** as a stable product.

### Cristallographic investigation

**Figure 3:**
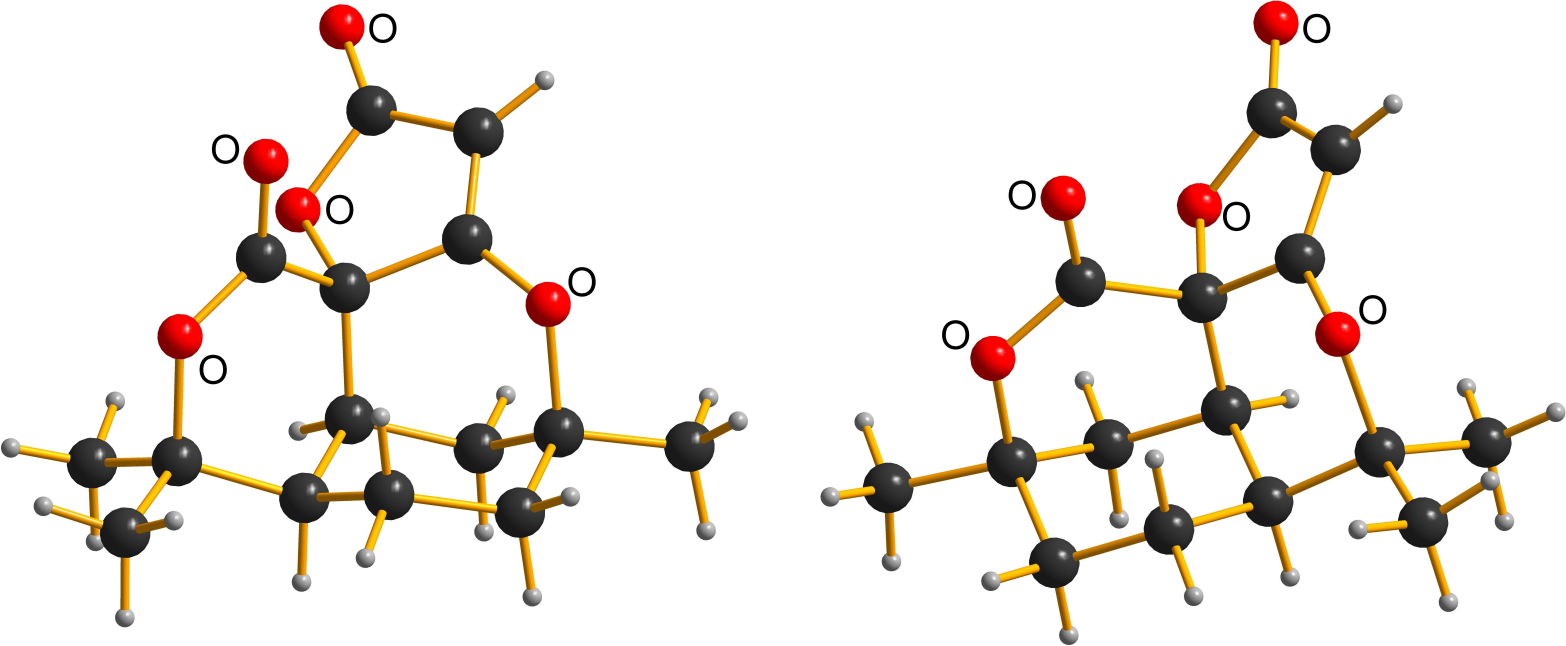
Left Part: **11** (main fraction); Right part: **12** (side product).

A problem arose during the X-ray crystallography of **11**. In addition to **11** DMF and an isomeric compound **12** were identified in the unit cell (Figure 3). Single-crystal X-ray diffraction data were collected for all samples using a Synergy diffractometer (Rigaku Oxford Diffraction) equipped with a photon-counting detector system [15]. Despite careful selection and mounting of the crystals, all specimens exhibited weak and diffuse diffraction patterns, which significantly limited data quality. This behavior is attributed to poor crystallinity and partial disorder within the crystal, likely exacerbated by the gradual release of solvent molecules during sample handling and data acquisition. Data collection was therefore applied with seriously elongated exposure time.

The crystal structure was successfully solved using methods as implemented in the SHELXT program [16], followed by refinement with SHELXL [17]. The refined asymmetric unit was found to contain about 2.75 molecules of compound **11** and 0.25 molecules of compound **12**, indicating partial occupancy between the two components in terms of a solid solution. This effect is caused by the similarity of the shape of the two molecules (see Figure 3). In addition, one molecule of DMF, presumably incorporated during crystallization, was identified within solvent-accessible voids in the crystal lattice. Due to its high level of disorder, the DMF molecule was removed computationally using the SQUEEZE routine implemented in the PLATON software package [18].

The final structural model clearly establishes the connectivity and constitution of both compounds within the crystal, despite the challenges posed by disorder and weak diffraction data. Structural parameters are consistent with the proposed molecular structures and support the presence of both components within a single crystalline phase.

Crystallographic data for the structural analysis have been deposited with the Cambridge Crystallographic Data Centre (CCDC) under deposition number 2477299. Copies of the data can be obtained free of charge via https://www.ccdc.cam.ac.uk/data_request/cif.

**Scheme 4:**
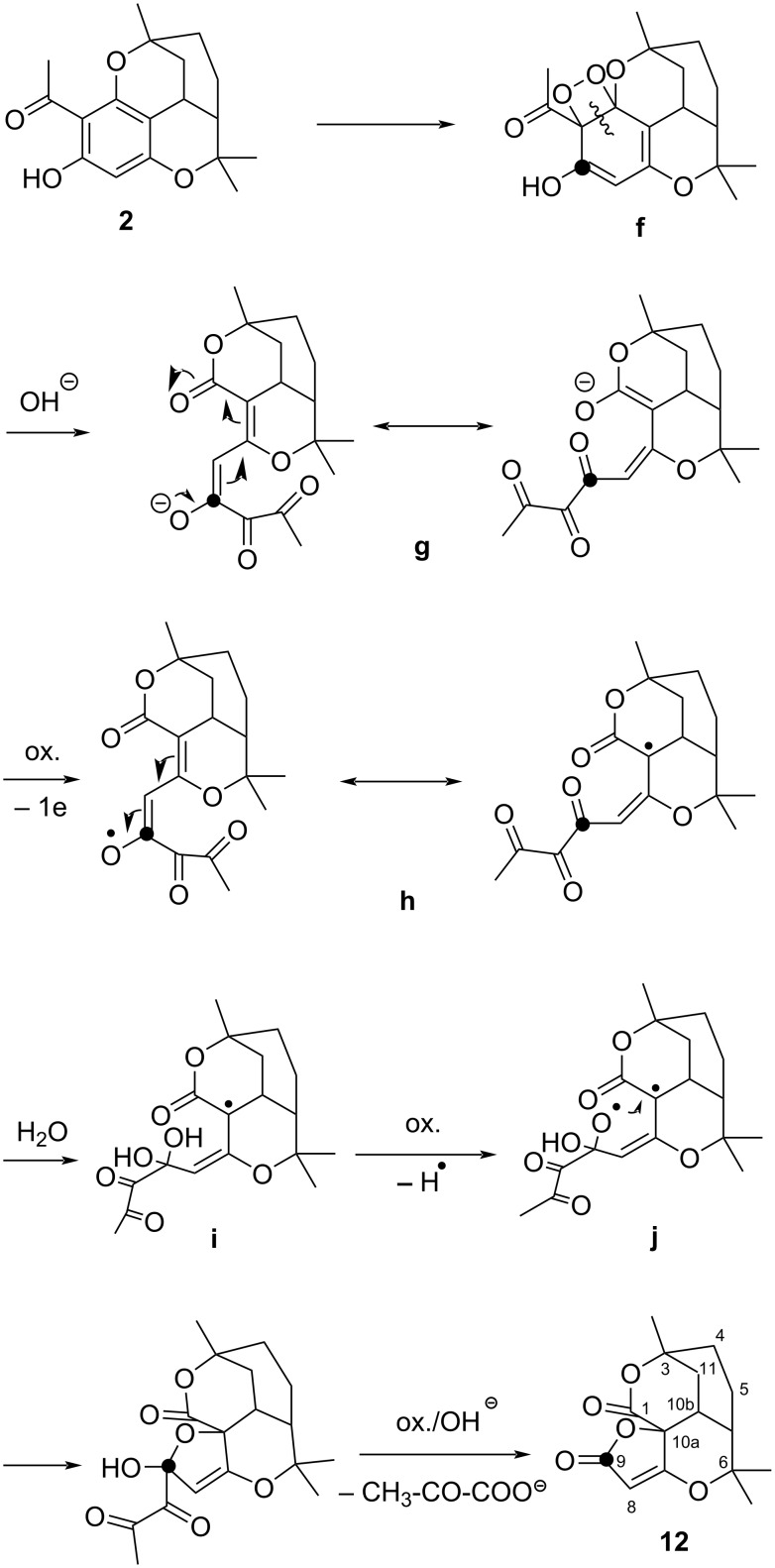
Oxidative ring contraction of melifolione B (**2**).

Compound **12** is formed by oxidative contraction of the acetylated phenol ring of melifolione B (**2**) which was present in the starting material (Scheme 4). Indeed, upon close inspection of the ^13^C NMR-spectrum of **11**, low intensity satellites were detected next to the high-field signals, while the deep-field signals were completely uniform. This fact was initially surprising, but can be explained based on the X-ray data. Both structures **11** and **12** can be precisely superimposed in the region of the furanone and pyranone rings (Figure 3). Therefore, the corresponding C-atoms have the same magnetic environment and the signals >100 ppm have no satellites. In contrast, the two cyclohexane rings of **11** and **12** are in a staggered position and their C-atoms are not magnetically equivalent. Due to the small amount of **12**, its C-signals appear as small satellites next to the intense signals of **11** in the NMR spectrum (see Supporting Information File 1, Figure S 23). In contrast, no clear evidence of signals from the isomer **12** could be detected in the ^1^H NMR spectrum of **11**.

**Scheme 5:**
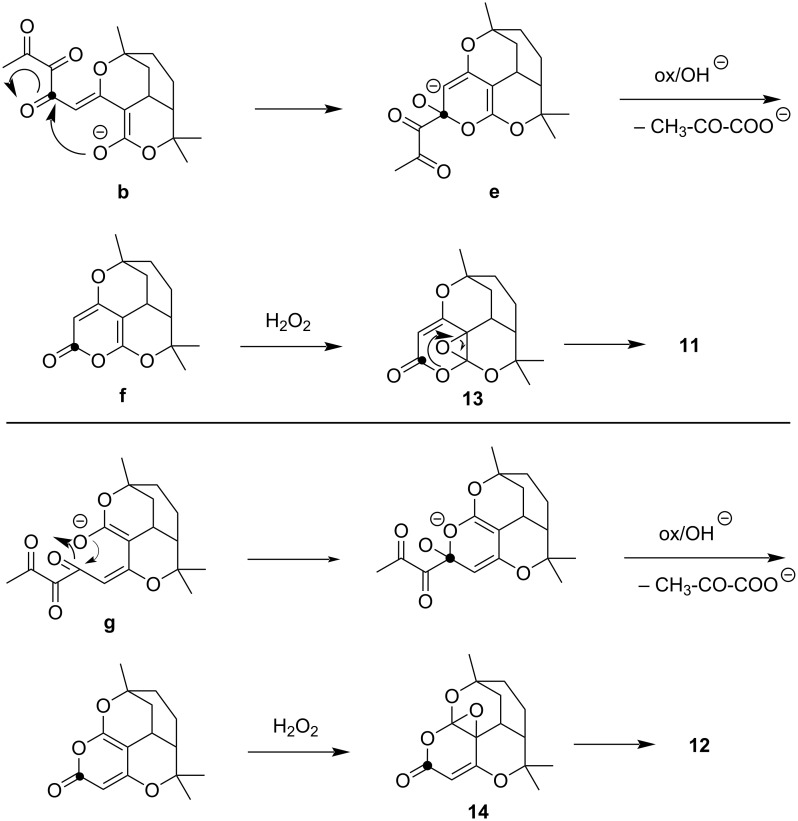
Alternative routes to the spirofuranones **11** and **12** via hypothetical pyranone epoxides **13** and **14**.

Oxidative ring contraction of melifolione B (**2**) can be explained in a similar way as in the case of melifolione A (**1**) (Scheme 4). Alternatively, the routes via intermediately formed pyranone epoxides **13** and **14** seems also to be possible [19] (Scheme 5).

Compounds **11** and **12** are the first representatives of two new heterocyclic ring systems consisting of two pyran rings, one cyclohexane and one furan ring.

## Conclusion

Finally, it remains to be noted that it was not possible to prepare the quinols **3** and **4** from mixtures of melifoliones **1** and **2** using the applied chemical methods. Thus, it is clearly proven, that **4** cannot be built as an artifact during the isolation. Therefore, it is likely that the biosynthesis of the quinol **4** occurs via enzymatically catalysed oxidation in the leaves of *Melicope barbigera*, where melifolione B (**2**) is present in higher concentrations than melifolione A (**1**) [1]. The isomeric melifoliones can be oxidized with hydrogen peroxide/ferricyanide in alkaline solution to give compounds **11** and **12** with new heterocyclic ring systems, formed by oxidative transformation of an acylated phenol into a furanone ring.

The result of this work was not expected and totally surprising. We not only present melifoliox B (**4**) as a new natural product but also found two new synthetic compounds with new heterocyclic ring systems as oxidation products of melifoliones A and B. Both structures could be clarified by NMR spectra and complex X-ray-analysis of a mixture of structural isomers in the elementary cell.

The importance of our results lies in the fact that it could open the door for future attempts to achieve enzymatically catalysed oxidation of the isomeric melifoliones to obtain the corresponding para quinols. Furthermore, the newly observed oxidative ring contraction of phloroacetophenones to furanone derivatives opens the possibility of more intensive investigation in this reaction with other suitable starting materials to explore scope and limitation of this reaction. This could lead to the elucidation of the reaction mechanism and the synthesis of new compounds with possible biological activity.

## Experimental

### General

Detailed experimental procedures are described in [1].

### Instrumentation

NMR spectra were recorded on Bruker ARX 300 or AVANCE DMX 600 NMR spectrometers (Bruker, Karlsruhe, Germany). High resolution mass spectra were recorded on a FTHRMS-Orbitrap mass spectrometer (Thermo-Finnigan, Waltham, MA, USA). Merck MN silica gel 60 M (0.04–0.063 mm) was used as stationary phase for column chromatography. TLC was performed on silica gel 60 F_254_ plates with UV-detection. Solvents and reagents were of analytical grade. Melting points were determined on a Büchi SMP 20. Microwave experiments were performed in sealed tubes on a CEM, model DISCOVER, power 300 Watt apparatus with a special temperature programme.

### Isolation of compound **4**

Compound **4** was isolated from the VLC-fraction MB-IX-5-P1 of the dichloromethane extract of *Melicope barbigera* leaves as a colorless and amorphous substance in an amount of 3.4 mg. *f*_R_ = 0.58 on silica 60 F_254_-plates (Merck) with hexane/dichloromethane/methanol 5:4:1.

**2-Acetyl-6a,7,8,9,10,10a-hexahydro-10b-hydroxy-6,6,9-trimethyl-1,9-epoxy-6*H*-dibenzo[*b*,*d*]pyran-3(10b*H*)-one (4):** UV (MeOH) l_max_ 250 nm; NMR data see Table 1 and Table 2; HRESIMS *m/z*: [M + H]^+^ calcd. for C_18_H_23_O_5_, 319.1540; found, 319.1542.

### Chemical synthesis of compounds

**1-[5,7-Dihydroxy-2-methyl-2-(4-methyl-3-pentenyl)-2*H*-1-benzopyran-8-yl]ethanone (5):** A mixture of 2,4,6-trihydroxyacetophenone (3.7 g, 20 mmol), citral (3.2 g, 21 mmol) and pyridine (1.6 g, 20 mmol) was heated with stirring in a water bath at max. 60 °C for 8 h. After cooling to room temperature, the reaction mixture was diluted with 200 mL diethyl ether and extracted with 0.1 N H_2_SO_4_ (3 × 20 mL) to remove the pyridine. Successive extraction with 0.1 N NaOH (5 × 20 mL) afforded a clear alkaline water phase that was acidified with dilute sulfuric acid and extracted with diethyl ether (5 × 20 mL). The organic phase was washed with water, dried over MgSO_4_, filtered and evaporated under reduced pressure to give 3,1 g (51.2%) of **5** (CAS: 59582-20-6) [20] as a beige colored viscous oil, which was used without further purification. HRESIMS *m/z*: [M + H]^+^ calcd. for C_18_H_23_O_4_, 303.1591; found, 303.1596. ^1^H NMR data in CDCl_3_ (δ ppm) 1.43 (s, 3H, 2-CH_3_), 1.56 (s, 3H, 4’-CH_3_), 1.67 (s, 3H, 4’-CH_3_), 1.70/1.85 (m, 2H, H-2’), 2.10/2.15 (m, 2H, H-1’), 2.66 (s, 3H, CH_3_CO), 5.10 (“t”, 1H, H-3’), 5,38 (d, *J* = 10.0 Hz, 1H, H-3), 5.96 (s, 1H, H-6), 6.61 (d, *J* = 10.0 Hz, 1H, H-4), 8.0 (br. s, 1H, 5-OH), 13.8 (s, 1H, 7-OH).

**Melifolione A (1) and melifolione B (2):** Chromene **5** (3 g, 10 mmol) was mixed with 5 drops of acetic acid in a sealed tube and heated in a microwave apparatus (CEM, model DISCOVER, power 300 Watt) up to 140 °C for 20 minutes. After cooling to room temperature, the reaction mixture was dissolved in 200 mL diethyl ether. This solution was washed with 0.1 N NaOH (3 × 20 mL) and water (1 × 20 mL), dried over MgSO_4_, filtered and evaporated to dryness. The remaining residue was recrystallized from diethyl ether/hexane to give 2.3 g (77%) colorless crystals, containing about 90% melifolione A (**1**) (CAS: 59779-71-4) and 10% melifolione B (**2**) (CAS: 37464-54-3), based on the ^1^H NMR spectrum. NMR data see Table 1 and Table 2.

**1-[3,4,5,6-Tetrahydro-7,9-dihydroxy-2-methyl-5-(1-methylethenyl)-2,6-methano-2*H*-1-benzoxocin-10-yl]ethanone (6):** Chromene **5** (910 mg, 3 mmol) was dissolved in 6 g acetic acid and heated on a water bath to 80 °C for 1 h. The reaction mixture was alkalized under cooling to pH 13 with 1 N NaOH (110 mL) and extracted with diethyl ether (3 × 20 mL). The remaining aqueous layer was acidified using 0.1 N H_2_SO_4_ and extracted with diethyl ether (3 × 30 mL). The organic phase was washed with NaHCO_3_ solution (1%) and water, dried with MgSO_4_, filtered and evaporated to give 520 mg (57%) of **6** (CAS: 66050-33-7) as colorless crystals from ethyl acetate/hexane, mp 181–182 °C [6]. HRESIMS *m/z*: [M + H]^+^ calcd. for C_18_H_23_O_4_ 303.1591; found, 303.1593; ^1^H NMR data in CDCl_3_ (δ ppm) 1.36 (s, 3H, 2-CH_3_), 1.47 (m, 2H, H_2_-4), 1.56 (m, 1H, H-3), 1.77 (dt, 1H, H-2’), 1.83 (s, 3H, 5’-CH_3_), 1.93 (dd, 1H, H-2’), 2.00 (dq, 1H, H-3), 2.30 (m, 1H, H-5), 2.62 (s, 3H, CH_3_CO), 3.38 (“q”, 1H, H-6), 4.51 (s, 1H, H-5’), 4.76 (s, 1H, H-5’), 5.83 (s, 1H, H-8), 10.5 (br. s, 1H, 7-OH), 13.7 (br. s, 1H, 9-OH); ^13^C NMR data in CDCl_3_ (δ ppm) 22.7 (C-4), 22.8 (5’-CH_3_), 28.7 (2-CH_3_), 31.3 (C-6), 32.9 (CH_3_CO), 37.7 (C-2’), 39.2 (C-3), 48.2 (C5), 76.2 (C-2), 95.0 (C-8), 103.2 (C-6a), 104.6 (C-10), 110.5 (C-5’’), 149.1 (C-5’), 160.5 (C-9), 162.0 (C-10a), 163.3 (C-7), 203.4 (CH_3_CO).

**1-[3,4,5,6-Tetrahydro-7,9-dihydroxy-2-methyl-5-(1-methylethylidene)-2,6-methano-2*H*-1-benzoxocin-10-yl]ethanone (7):** Chromene **5** (910 mg, 3 mmol) was dissolved in toluene (50 mL) and 4-toluenesulfonic acid (50 mg) was added. The mixture was heated under stirring on a water bath at 80 °C for 3 h. After cooling to room temperature, diethyl ether (100 mL) was added, and the organic phase was extracted with 0.1 N NaOH (3 × 30 mL). The alkaline aqueous layer was acidified with 0.1 N H_2_SO_4_ and extracted with diethyl ether (3 × 30 mL). The ether phase was washed with NaHCO_3_ solution (1%) and water, dried over MgSO_4_, filtered and evaporated under reduced pressure to give 615 mg (68%) **7** (CAS: 37463-56-2) as white solid, which was recrystallized from diethyl ether/hexane, mp 222 °C [6]. HRESIMS *m/z*: [M + H]^+^ calcd. for C_18_H_23_O_4_, 303.1591; found, 303.1593; ^1^H NMR data in CDCl_3_ (δ ppm) 1.43 (s, 3H, 2-CH_3_), 1.58 (m, 1H, H-2’), 1.66 (s, 3H, 5’-CH_3_), 1.83 (m, 2H, H-3), 1.89 (s, 3H, 5’-CH_3_), 1.90 (m, 1H, H-4), 2.03 (dd, 1H, H-2’), 2.45 (dd, 1H, H-4), 2.65 (s, 3H, CH_3_CO), 4.16 (“t”, 1H, H-6), 5.4 (s, 1H, 7-OH), 5.85 (s, 1H, H-8), 13.7 (s, 1H, 9-OH); ^13^C NMR data in CDCl_3_ (δ ppm, */** exchangeable) 20.1 (5’-CH_3_), 20.8 (5’-CH_3_), 22.8 (C-4), 28.8 (2-CH_3_), 29.6 (C-6), 33.5 (CH_3_CO), 36.6 (C-3), 40.6 (C-2’), 66.0 (C-2), 95.2 (C-8), 104.1 (C-6a*), 105.6 (C-10*), 122.5 (C-5’), 131.2 (C-5), 159.4 (C-9**), 159.9 (C-10a**), 164.9 (C-7), 203.6 (CH_3_CO).

**Iodized derivatives of melifolione A (8) and melifolione B (9):** A mixture of melifolione A (**1**) and B (**2**) (60 mg, 0.2 mmol, ratio ca.10:1) was dissolved in DMF (2 mL) and 1 N NaOH (5 mL) was added. Lugol’s solution (10 mL of 1% iodine/potassium iodide in water) was added dropwise while cooling with ice and stirring. After 30 minutes, the suspension was diluted with water (80 mL), acidified with 0.1 N H_2_SO_4_ and mixed with aqueous solution of Na_2_S_2_O_3_ (1%) until discoloration. After usual work up, the ethyl acetate extract (3 × 20 mL) yielded 76 mg (88%) of a mixture of 2-iodomelifolione A (**8**) and 4-iodomelifolione B (**9**) in a ratio of 10:1 as white solid from diethyl ether/hexane. TLC on silica gel 60 F_254_ plates with hexane/ethyl acetate (10+2): *f*_R_ = 0.28 in relation to melifoliones *f*_R_ = 0.38. HRESIMS *m/z*: [M + H]^+^ calcd. for C_18_H_22_IO_4_, 429.0557; found, 429.0564; ^1^H NMR spectra revealed the relation of both isomers in the same ratio as in the educts. Significant signals were separated and could be assigned because of their intensity (**8**:**9** = ca. 10:1).

**2-Iodomelifolione A (8):**^1^H NMR data in CDCl_3_ (δ ppm) 0.85 (m, 1H, H-7), 1.13 (s, 3H, 6-CH_3_), 1.33 (quint, 1H, H-7), 1.50 (s, 3H, 9-CH_3_), 1.52 (m, 1H, H-8), 1.60 (s, 3H, 6-CH_3_), 1.86 (m, 1H, 8-H), 1.89 (dd, 1H, H-10), 2.10 (ddd, 1H, H-6a), 2.21 (ddd, 1H, H-10), 2.65 (s, 3H, CH_3_CO), 2.75 (“s”, 1H, H-10a), 14.4 (s, 1H, OH); ^13^C NMR data in CDCl_3_ (δ ppm, *no difference between **8** and **9**) 21.8 (C-7), 24.5 (6-CH_3_), 28.0 (C-10a), 28.5 (9-CH_3_), 30.0 (6-CH_3_), 31.8 (CH_3_CO), 34.9 (C-10), 37.6* (C-8), 46.09 (C-6a), 65.3 (C-2), 77.9* (C-9), 87.1 (C-6), 107.6 (C-4), 108.0 (C-10b), 159.7 (C-4a), 161.6 (C-1), 162.8 (C-3), 202.0 (CH_3_CO).

**4-Iodomelifolione B (9):**^1^H NMR data in CDCl_3_ (δ ppm) only the following signals were significantly separated from the corresponding signals of **8** and could be assigned to **9**: 1.16 (s, 6-CH_3_), 1.45 (s, 9-CH_3_), 1.62 (s, 6-CH_3_), 2.85 (“s”, H-10a), 14.7 (s, OH); ^13^C-NMR data in CDCl_3_ (δ ppm, *no difference between **8** and **9**) 21.9 (C-7), 24.7 (6-CH_3_), 28.1 (C-10a), 28.7 (9-CH_3_), 29.6 (6-CH_3_), 32.1 (CH_3_CO), 34.4 (C-10), 37.6* (C-8), 46.11 (C-6a), 66.3 (C-4), 77.9* (C-9), 87.4 (C-6), 107.0 (C-2), 107,4 (C-10b), 159.3 (C-4a), 162.5 (C-1), 163.6 (C-3), 202.4 (CH_3_CO).

**2-Iodo-3-phenoxymelifolione A (10):** In analogy to a literature procedure [21], a mixture of melifolione A (**1**) and B (**2**) (181 mg, 0.6 mmol, ratio ca. 10:1) was dissolved in 16 mL acetonitrile. After addition of water (4 mL), the solution was cooled on ice and iodosobenzene diacetate (212 mg, 0.66 mmol) was added under stirring. The solution turned yellow and later orange. TLC control showed consumption of the educts after 30 min. Saturated aqueous solutions of Na_2_S_2_O_3_, Na_2_CO_3_ and NaCl (each 20 mL) were added together with ethyl acetate (50 mL) to generate two phases. After separation of the organic layer and subsequent extraction of the aqueous phase with ethyl acetate (3 × 20 mL), the organic layer was dried over MgSO_4_, filtered and evaporated under reduced pressure. The residue was chromatographed on silica gel with hexane/ethyl acetate (5:1 to 1:1) to yield 55 mg (18%) colorless solid. TLC on silica gel 60 F_254_ plates with hexane/ethyl acetate (10:1): *f*_R_ = 0.15 in relation to melifoliones *f*_R_ = 0.25. HRESIMS *m/z*: [M + H]^+^ calcd. for C_24_H_26_IO_4_, 505.0870; found, 505.0875; ^1^H NMR data in CDCl_3_ (δ in ppm) 0.87 (m, 1H, H-7), 1.05 (s, 3H, 6-CH_3_), 1.31 (m, 1H, H-7), 1.49 (s, 3H, 9-CH_3_), 1.50 (m, 1H, H-8), 1.55 (s, 3H, 6-CH_3_), 1.87 (m, 1H, H-8), 1.91 (dd, 1H, H-10), 2.13 (ddd, 1H, H-6a), 2.26 (dt, 1H, H-10), 2.35 (s, 3H, CH_3_CO), 2.88 (“s”, 1H, H-10a), 6.75 (d, 2H, H-2’/H-6’), 6.97 (t, 1H, H-4’), 7.23 (t, 2H, H-3’/H-5’).

**Octahydro-1,1,6-trimethyl-3’,6-epoxy-spiro[1*H*-2-benzopyran-4,2’(5’*H*)-furan]-3,5’-dione (11):** A mixture of **1** and **2** (300 mg, 1 mmol, ratio ca. 10:1) was dissolved in 30 mL acetonitrile, cooled on ice, and 5.7 mL H_2_O_2_ solution 3% (5 mmol) was added. A solution of potassium ferricyanide (1.65 g, 5 mmol) in 20 mL of 0.5 N NaOH (10 mmol) was added dropwise under stirring while cooling on ice. The reaction mixture initially turned purple and later orange. Within 30 min. the starting material had disappeared (TLC). After adding 50 mL 0.1 N NaOH, the mixture was extracted with diethyl ether (3 × 30 mL). The organic phase was washed with water (3 × 10 mL), dried with MgSO_4_, filtered and evaporated under reduced pressure to give **11** as a colorless viscous oil (85 mg, 31%), which solidified after some time (mp 153–154°C). HRESIMS *m/z*: [M + H]^+^ calcd. for C_15_H_19_O_5_, 279.1227; found, 279.1226; NMR-data see Table 3. TLC on silica gel 60 F_254_ plates with hexane/dichloromethane/ethyl acetate (10:8:2): *f*_R_ = 0.15, compared to **1** and **2** with *f*_R_ = 0.48. For X-ray analysis, compound **11** was recrystallized from diethyl ether/hexane.

**Hexahydro-3,6,6-trimethyl-{3,5-ethano-9*H*-furo[3,2-*i*]1*H*,3*H*-pyrano[3,4-*c*]pyran}-1,9-dione (12):** ^13^C NMR data in CDCl_3_ (δ ppm): 19.49, 25.97, 26.04, 27.94, 32.62, 34.53, 35.90, 40.13, 80.14, 82.65, 87.41, 101.4*, 165.2*, 172.2*, 179.4*.*) no difference to compound **11**.

## Supporting Information

File 1 Copies of the HRESIMS, ^1^H and ^13^C NMR spectra.

## Data Availability

All data that supports the findings of this study is available in the published article and/or the supporting information of this article.
