# Supplementary material for: Melifoliox B, a novel phloroglucin derivative isolated from Melicope barbigera (Rutaceae) and synthesis of new oxidation products from melifoliones A and B
Source: Beilstein J Org Chem. 2026 Mar 24;22:535–46. doi: 10.3762/bjoc.22.39 (PMC13040265; doi:10.3762/bjoc.22.39)
Supplement: File 1 — Copies of the HRESIMS, 1H and 13C NMR spectra. [file Beilstein_J_Org_Chem-22-535-s001.pdf]

BEILSTEIN JOURNAL OF ORGANIC CHEMISTRY

Supporting Information

for

Melifoliox B, a novel phloroglucin derivative isolated from *Melicope barbigera* (Rutaceae) and synthesis of new oxidation products from melifoliones A and B

Horst Weber, Kim-Thao Tran-Cong, Bernhard Mayer, Guido J. Reiss,
Iryna S. Konovalova, Marc S. Appelhans, Kenneth R. Wood and Claus M. Passreiter

Beilstein J. Org. Chem. **2026,** *22,* 535–546. doi:10.3762/bjoc.22.39

Copies of the HRESIMS, ^{1}H and ^{13}C NMR spectra

License and Terms: This is a supporting information file under the terms of the Creative Commons Attribution License (https://creativecommons.org/licenses/by/4.0). Please note that the reuse, redistribution and reproduction in particular requires that the author(s) and source are credited and that individual graphics may be subject to special legal provisions.
The license is subject to the *Beilstein Journal of Organic Chemistry* terms and conditions: (https://www.beilstein-journals.org/bjoc/terms)

Table of content

Figure S1. HRESIMS of compound **1+2** S2

Figure S2 ^{1}H NMR (600 MHz, CDCl3) spectrum of compound **1** S3

Figure S3.^{13}C NMR (600 MHz, CDCl3) spectrum of compound **1** S3

Figure S4. ^{1}H NMR (600 MHz, $CDCl_3$) spectrum of compound **2** S4

Figure S5. ^{13}C NMR (150 MHz, $CDCl_3$) spectrum of compound **2** S4

Figure S6. HRESIMS of compound **4** S5

Figure S7. ^{1}H NMR (600 MHz, $CDCl_3$) spectrum of compound **4** S5

Figure S8. ^{13}C NMR (150 MHz, $CDCl_3$) spectrum of compound **4** S6

Figure S9. HRESIMS of compound **5.** S6

Figure S10. HRESIMS of compound **6**. S7

Figure S11. ^{1}H NMR (600 MHz, $CDCl_3$) spectrum of compound **6** S7

Figure S12. ^{13}C NMR (150 MHz, $CDCl_3$) spectrum of compound **6** S8

Figure S13. HRESIMS of compound **7.** S8

Figure S14. ^{1}H NMR (600 MHz, $CDCl_3$) spectrum of compound **7** S9

Figure S15. ^{13}C NMR (150 MHz, $CDCl_3$) spectrum of compound **7** S9

Figure S16. HRESIMS of compound **8+9.** S10

Figure S17. ^{1}H NMR (600 MHz, $CDCl_3$) spectrum of compound **8+9** S10

Figure S18. ^{13}C NMR (150 MHz, $CDCl_3$) spectrum of compound **8+9** S11

Figure S19. HRESIMS of compound **10**. S11

Figure S20. ^{1}H NMR (600 MHz, $CDCl_3$) spectrum of compound **10** S12

Figure S21. HRESIMS of compound **11** S12

Figure S22. ^{1}H NMR (600 MHz, $CDCl_3$) spectrum of compound **11** S13

Figure S23. ^{13}C NMR (150 MHz, $CDCl_3$) spectrum of compound **11** S13

Figure S24. 2D-COSY-spectrum of compound **11** S14

Figure S25. 2D-HSQC-spectrum of compound **11** S14

Figure S26. 2D-HMBC-spectrum of compound **11** S15

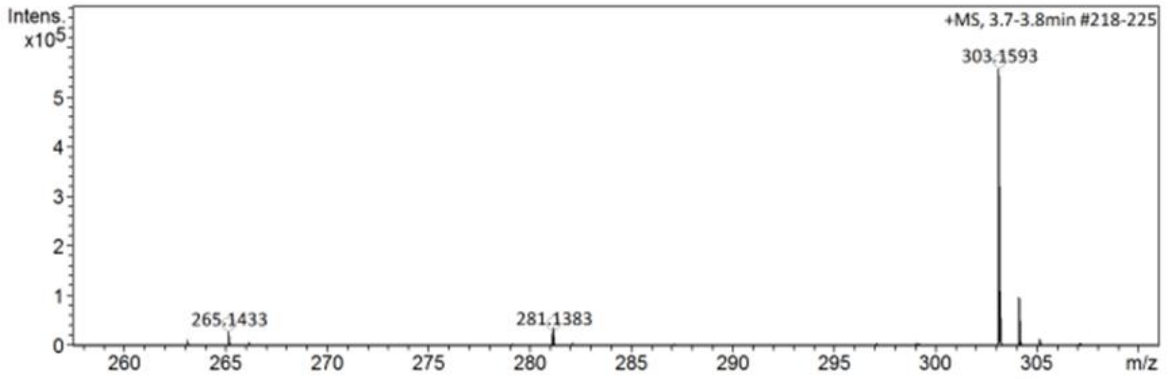

Meas. m/z	#	Ion Formula	m/z	err [ppm]	mSigma	# mSigma	Score	rdb	e⁻ Conf	N-Rule
265.1433	1	C15H21O4	265.1434	0.4	6.7	1	100.00	5.5	even	ok
	2	C16H17N4	265.1448	5.5	9.0	2	47.00	10.5	even	ok
281.1383	1	C15H21O5	281.1384	0.3	6.6	1	100.00	5.5	even	ok
	2	C16H17N4O	281.1397	5.1	20.4	2	37.71	10.5	even	ok
303.1593	1	C18H23O4	303.1591	-0.8	13.2	1	100.00	7.5	even	ok

Figure S1. HRESIMS of compound **1+2**.

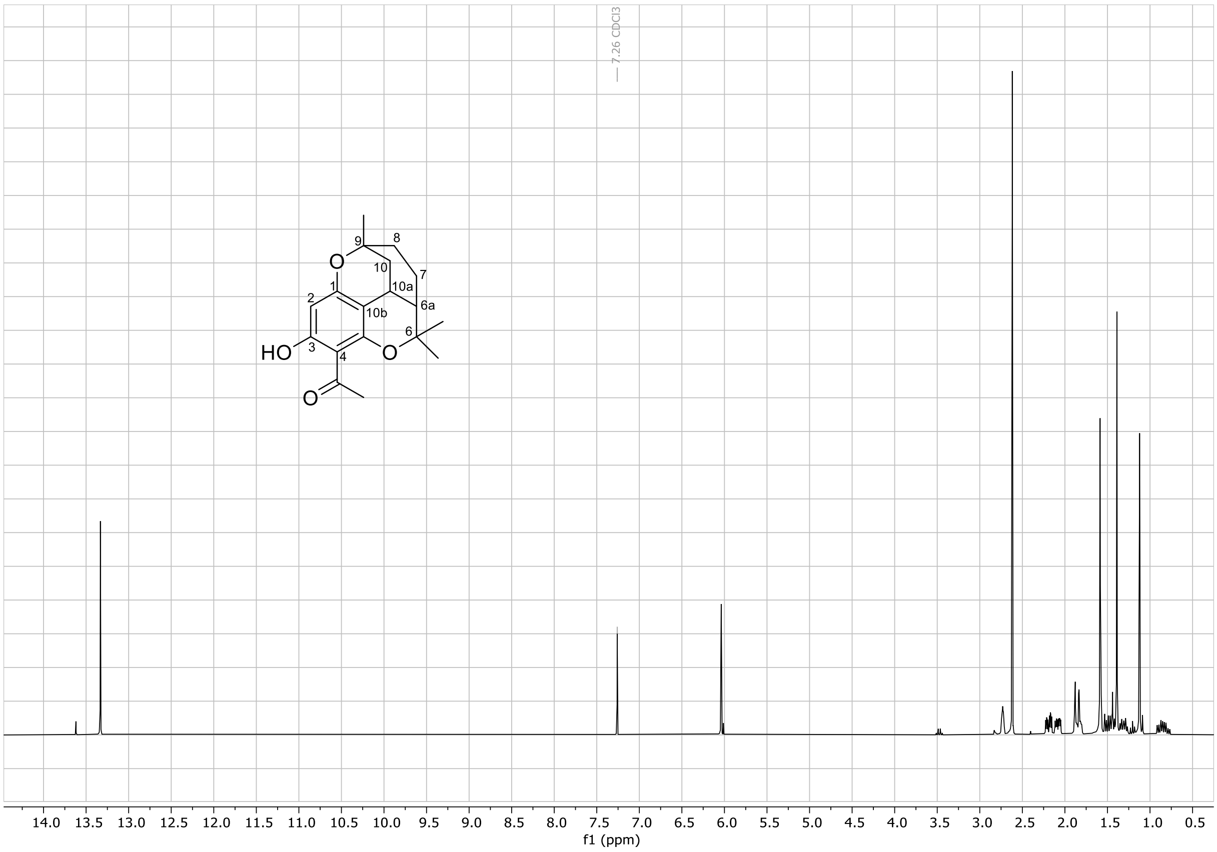

Figure S2. ^{1}H NMR (600 MHz, $CDCl_3$) spectrum of compound **1.**

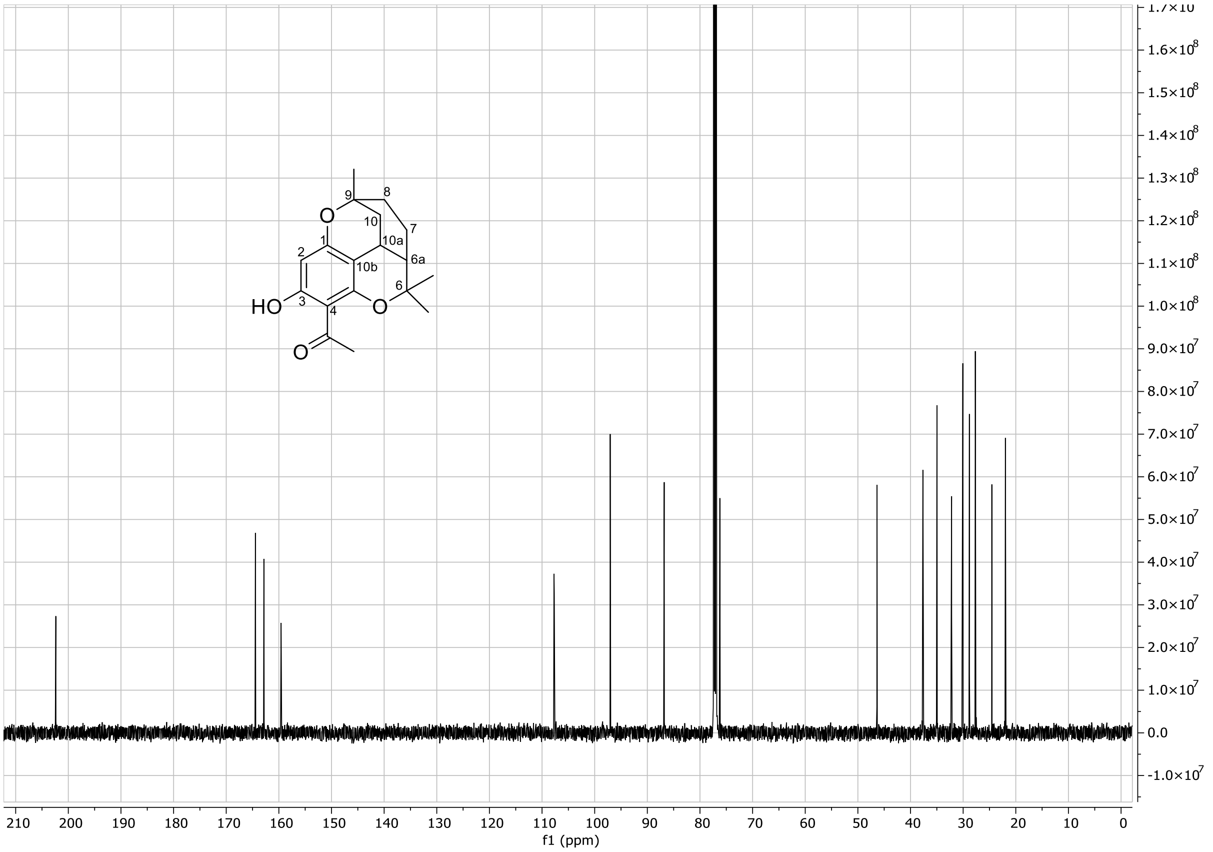

Figure S3. ^{13}C NMR (150 MHz, $CDCl_3$) spectrum of compound **1.**

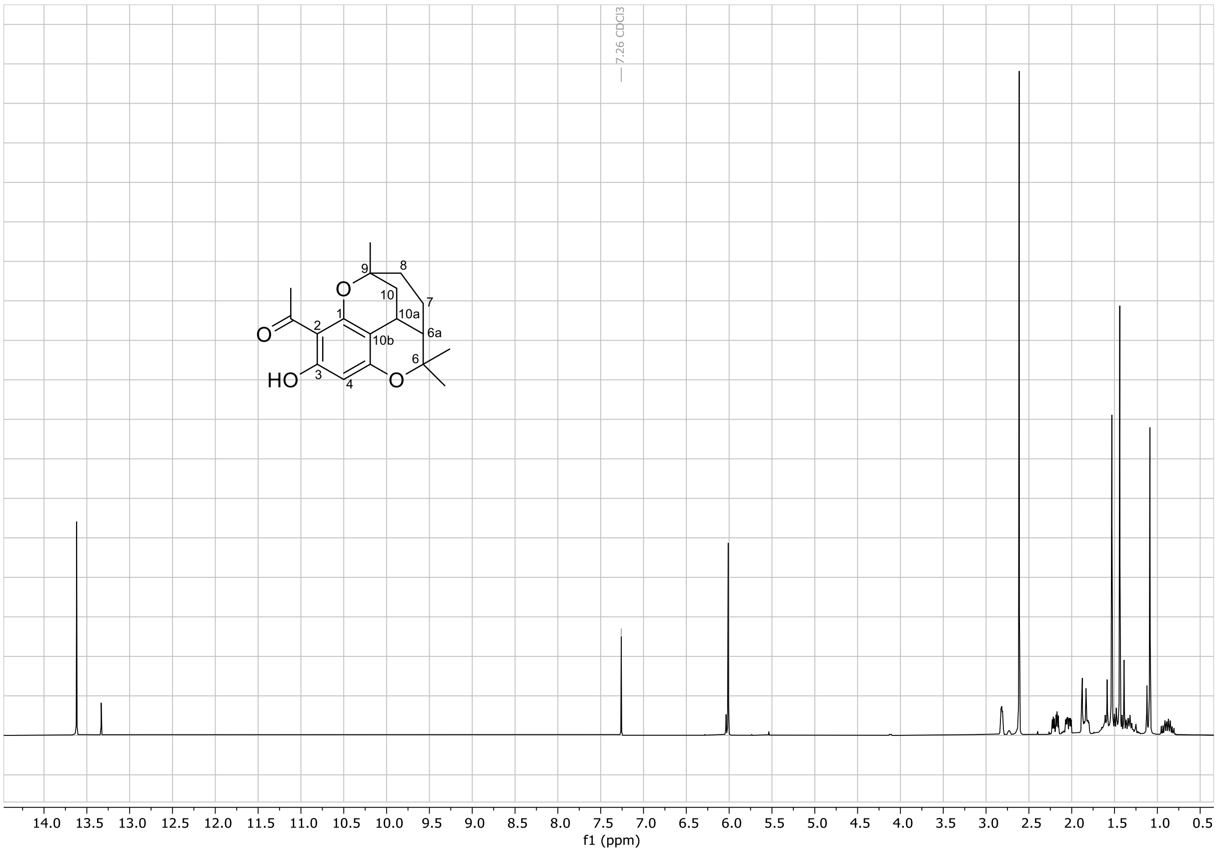

Figure S4. ^{1}H NMR (600 MHz, $CDCl_3$) spectrum of compound **2.**

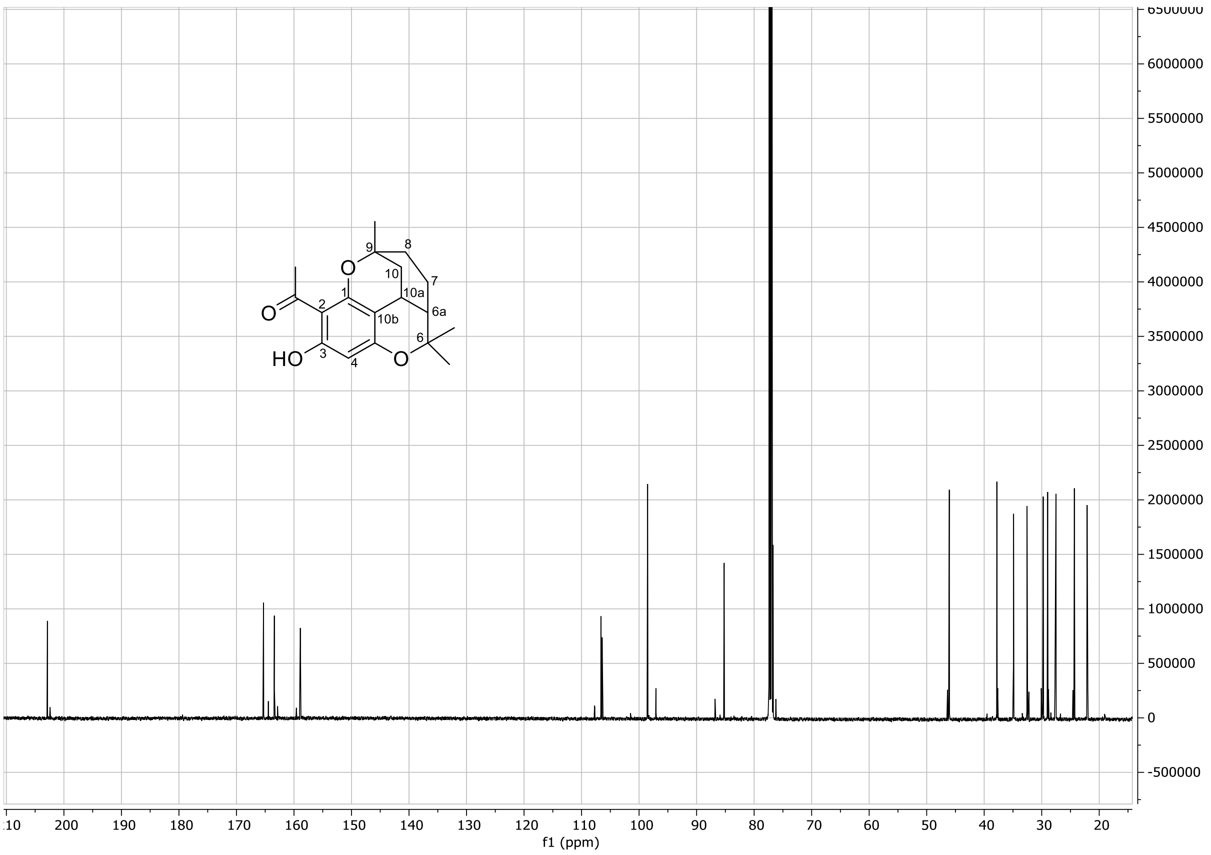

Figure S5. ^{13}C NMR (150 MHz, $CDCl_3$) spectrum of compound **2.**

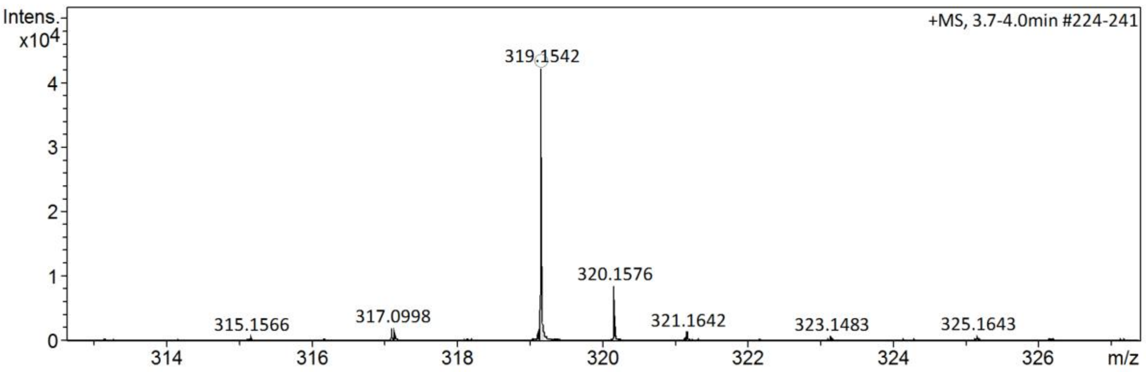

Meas. m/z	#	Ion Formula	m/z	err [ppm]	mSigma	# mSigma	Score	rdb	e⁻ Conf	N-Rule
319.1542	1	C18H23O5	319.1540	-0.6	0.8	1	100.00	7.5	even	ok
	2	C19H19N4O	319.1553	3.6	13.0	2	49.11	12.5	even	ok

Figure S6. HRESIMS of compound **4**.

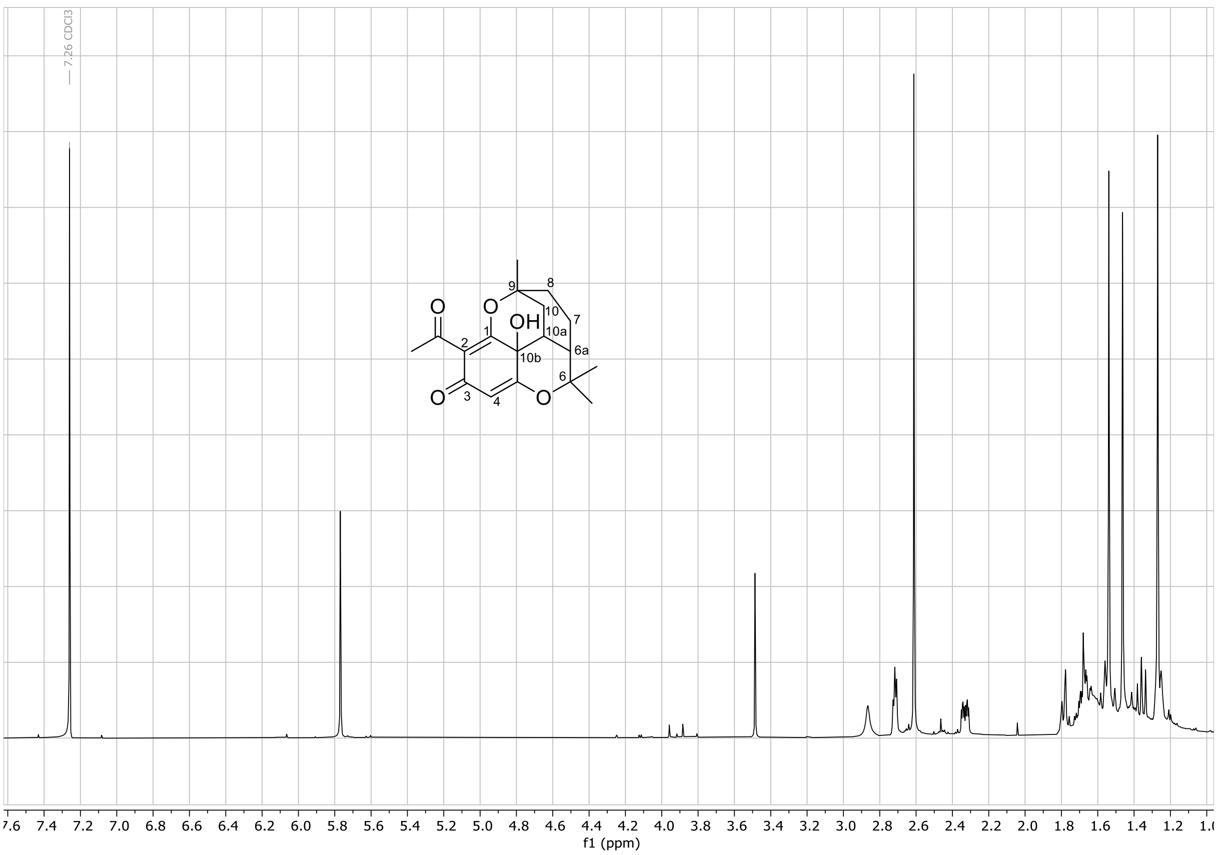

Figure S7. ^{1}H NMR (600 MHz, $CDCl_3$) spectrum of compound **4.**

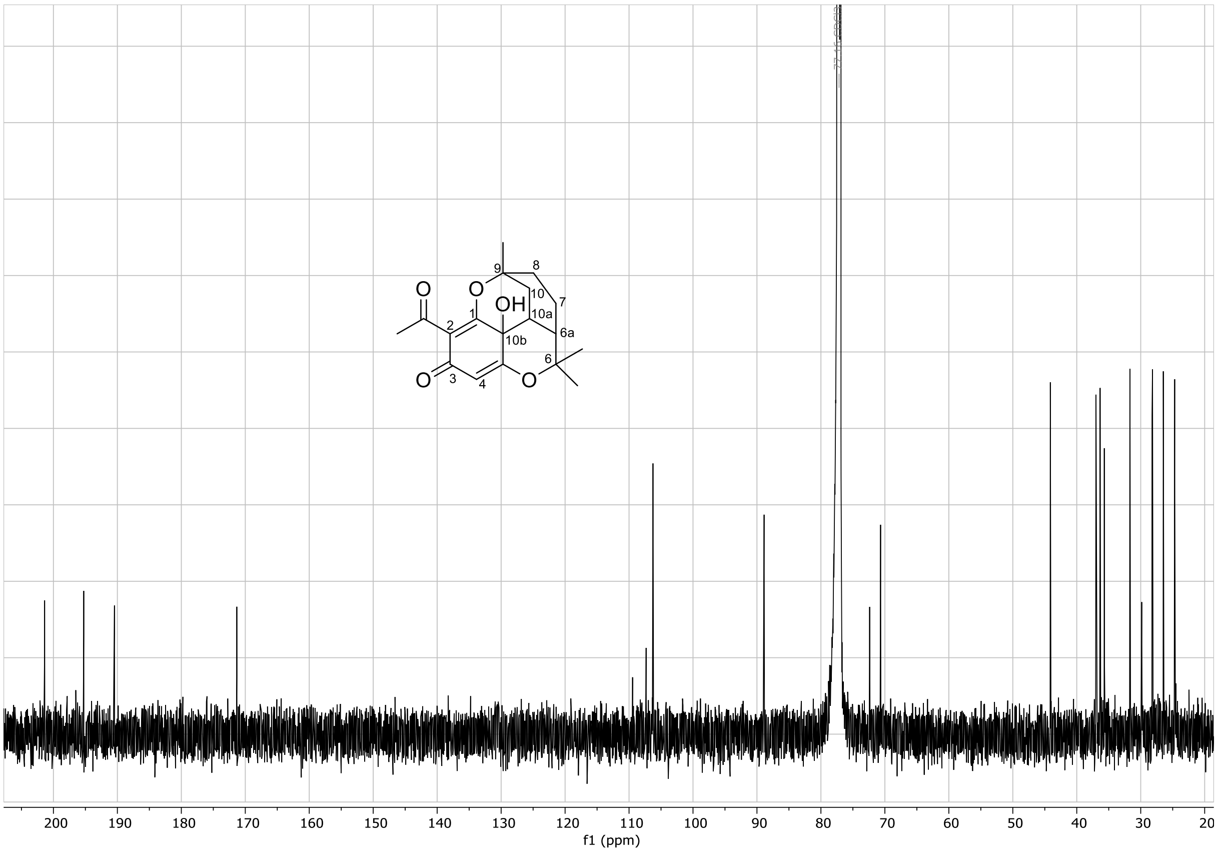

Figure S8. ^{13}C NMR (150 MHz, $CDCl_3$) spectrum of compound **4.**

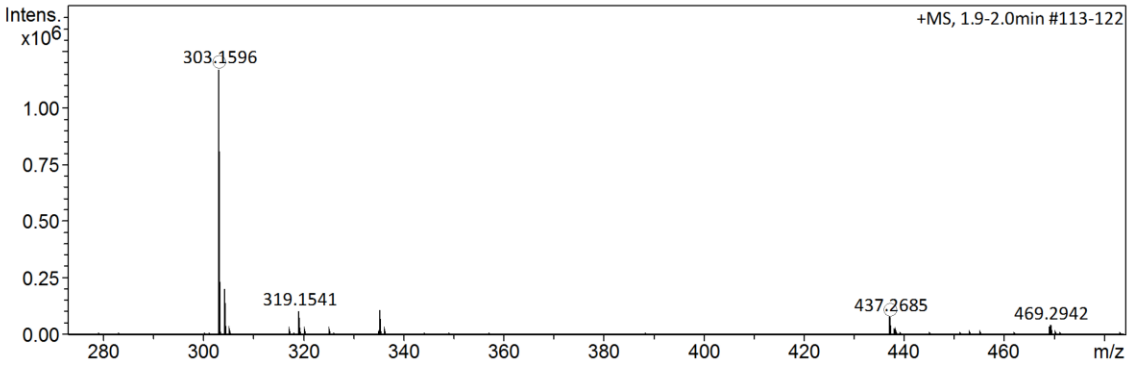

Meas. m/z	#	Ion Formula	m/z	err [ppm]	mSigma	# mSigma	Score	rdb	e⁻ Conf	N-Rule
303.1596	1	C18H23O4	303.1591	-1.9	15.7	1	100.00	7.5	even	ok
437.2685	1	C28H37O4	437.2686	0.3	4.5	1	100.00	10.5	even	ok

Figure S9. HRESIMS of compound **5.**

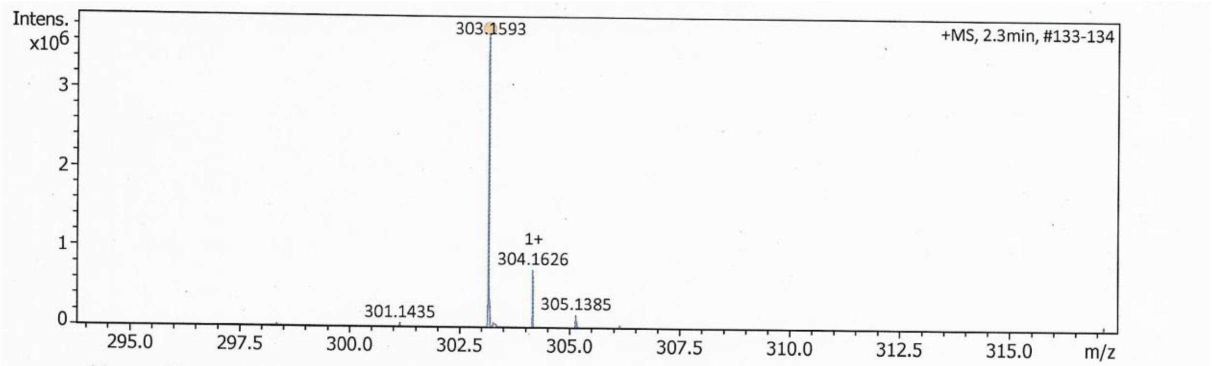

Meas. m/z	#	Ion Formula	m/z	err [ppm]	Mean err [ppm]	rdb (neutral)	N-Rule	e⁻ Conf	mSigma	Std I	Std Mean m/z	Std I VarNorm	Std m/z Diff	Std Comb Dev
303.1593	1	C18H23O4	303.1591	-0.8	-0.3	8.0	ok	even	2.7	5.3	n.a.	n.a.	n.a.	n.a.
303.1593	2	C19H19N4	303.1604	3.6	3.7	13.0	ok	even	15.7	25.4	n.a.	n.a.	n.a.	n.a.

Figure S10. HRESIMS of compound **6**.

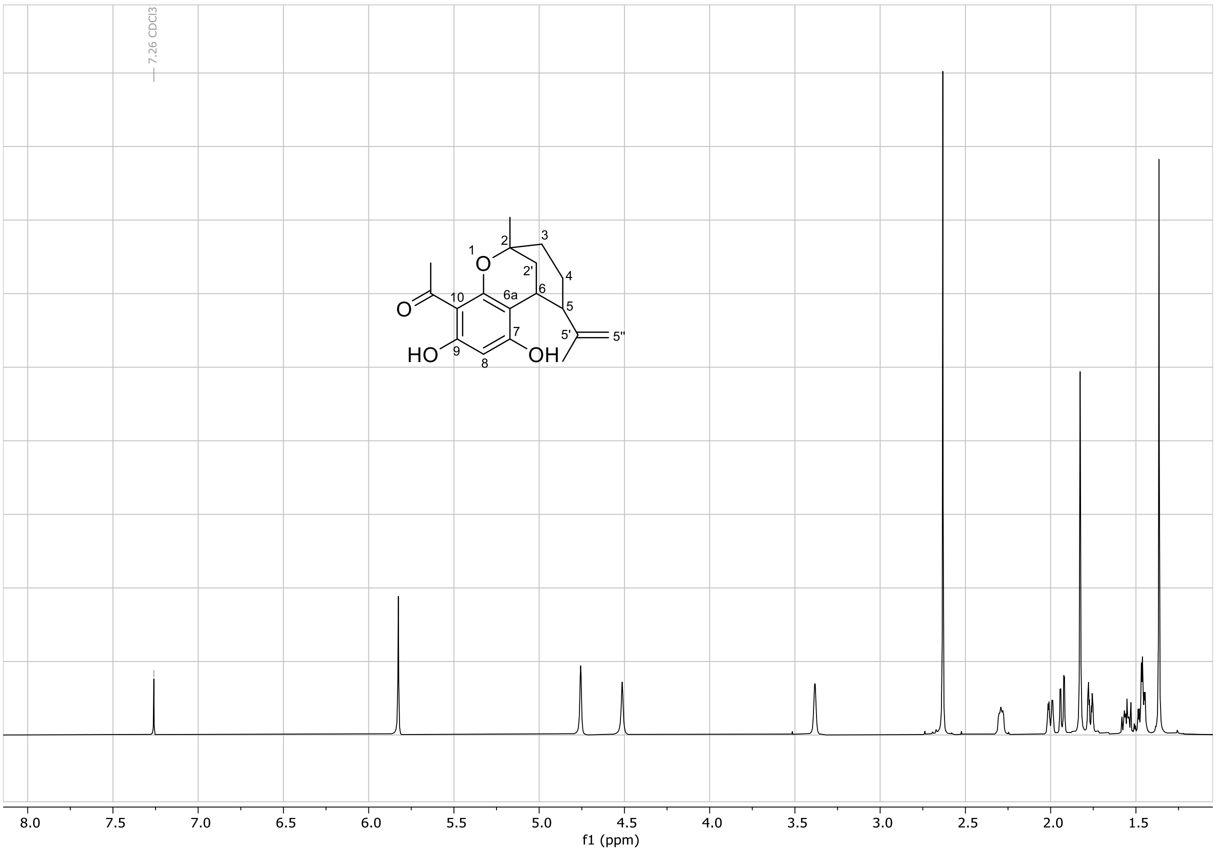

Figure S11. ^{1}H NMR (600 MHz, $CDCl_3$) spectrum of compound **6.**

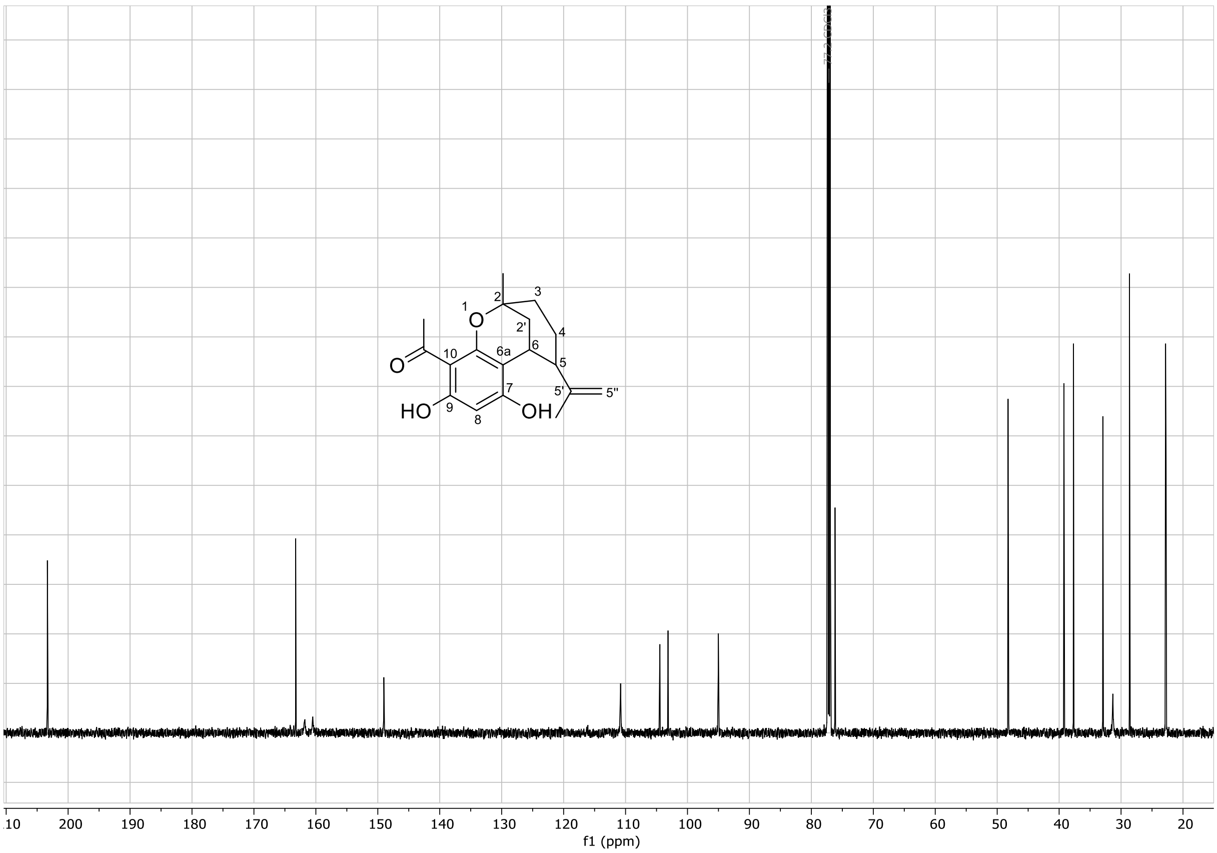

Figure S12. ^{13}C NMR (150 MHz, $CDCl_3$) spectrum of compound **6.**

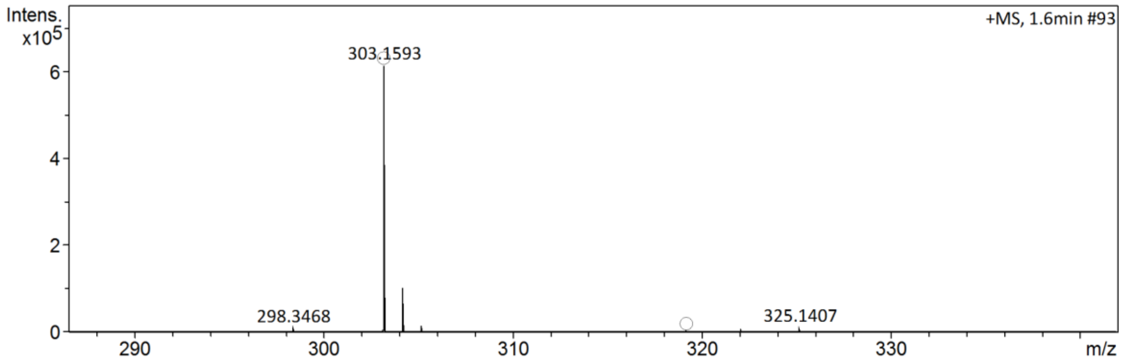

Meas. m/z	#	Ion Formula	m/z	err [ppm]	mSigma	# mSigma	Score	rdb	e⁻ Conf	N-Rule
303.1593	1	$C_{18}H_{23}O_4$	303.1591	-0.8	18.4	1	100.00	7.5	even	ok
	2	$C_{17}H_{20}N_4Na$	303.1580	-4.3	19.2	2	55.70	9.5	even	ok
	3	$C_{19}H_{19}N_4$	303.1604	3.6	31.6	3	47.68	12.5	even	ok
319.1537	1	$C_{19}H_{19}N_4O$	319.1553	5.1	15.0	1	55.73	12.5	even	ok
	2	$C_{17}H_{20}N_4NaO$	319.1529	-2.4	19.3	2	86.19	9.5	even	ok
	3	$C_{18}H_{23}O_5$	319.1540	0.9	23.2	3	100.00	7.5	even	ok

Figure S13. HRESIMS of compound **7**.

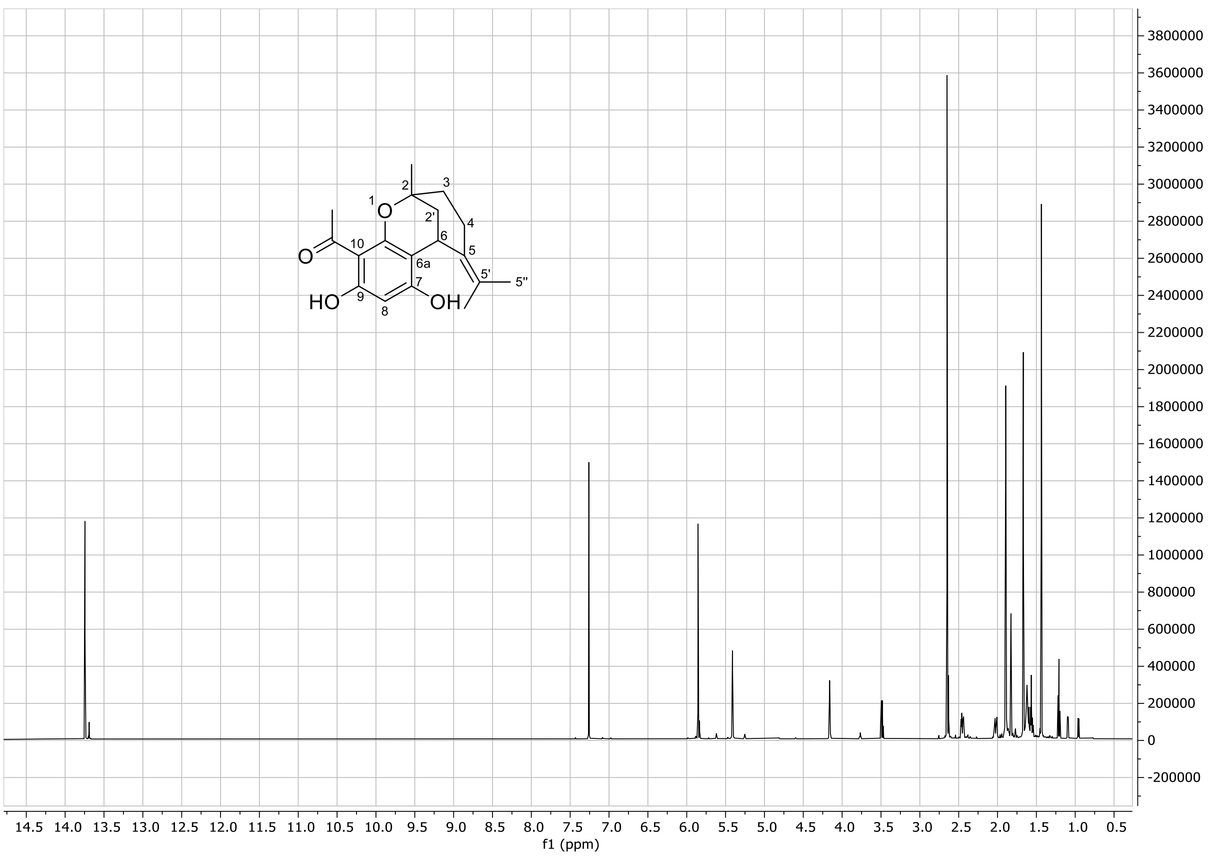

Figure S14. ^{1}H NMR (600 MHz, $CDCl_3$) spectrum of compound **7.**

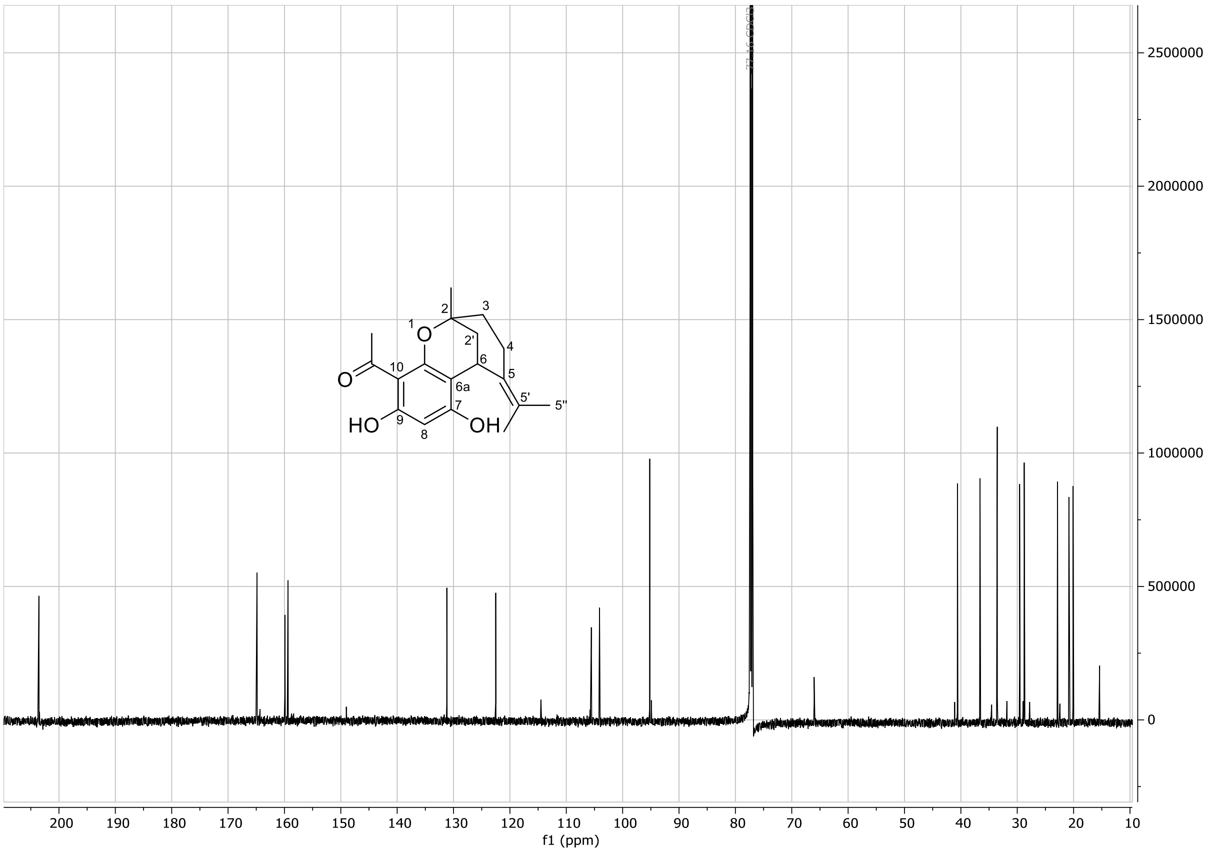

Figure S15. ^{13}C NMR (150 MHz, $CDCl_3$) spectrum of compound **7.**

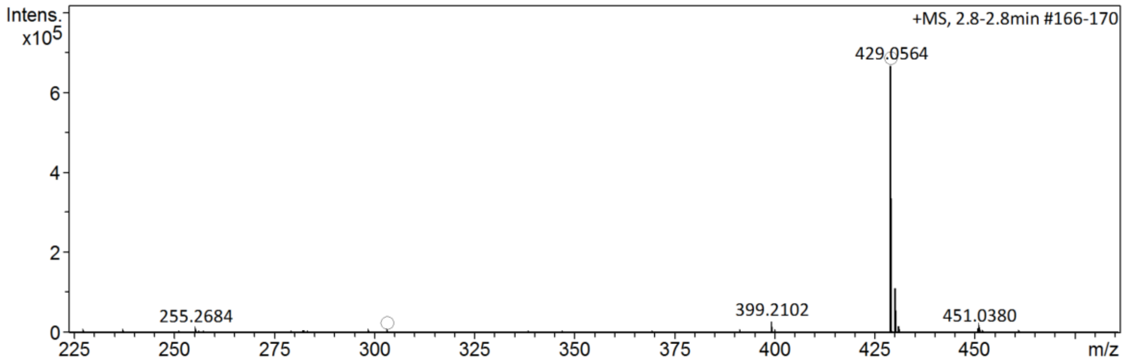

Meas. m/z	#	Ion Formula	m/z	err [ppm]	mSigma	# mSigma	Score	rdb	e¯ Conf	N-Rule
303.1591	1	C18H23O4	303.1591	-0.1	15.6	1	100.00	7.5	even	ok
	2	C19H19N4	303.1604	4.4	19.6	2	47.19	12.5	even	ok
429.0564	1	C18H22IO4	429.0557	-1.6	17.9	1	100.00	7.5	even	ok
	2	C19H18IN4	429.0571	1.5	31.2	2	74.15	12.5	even	ok
	3	C19H13N2O10	429.0565	0.2	31.3	3	97.57	14.5	even	ok
	4	C17HN16	429.0565	0.1	43.1	4	71.73	25.5	even	ok

Figure S16. HRESIMS of compound **8+9**.

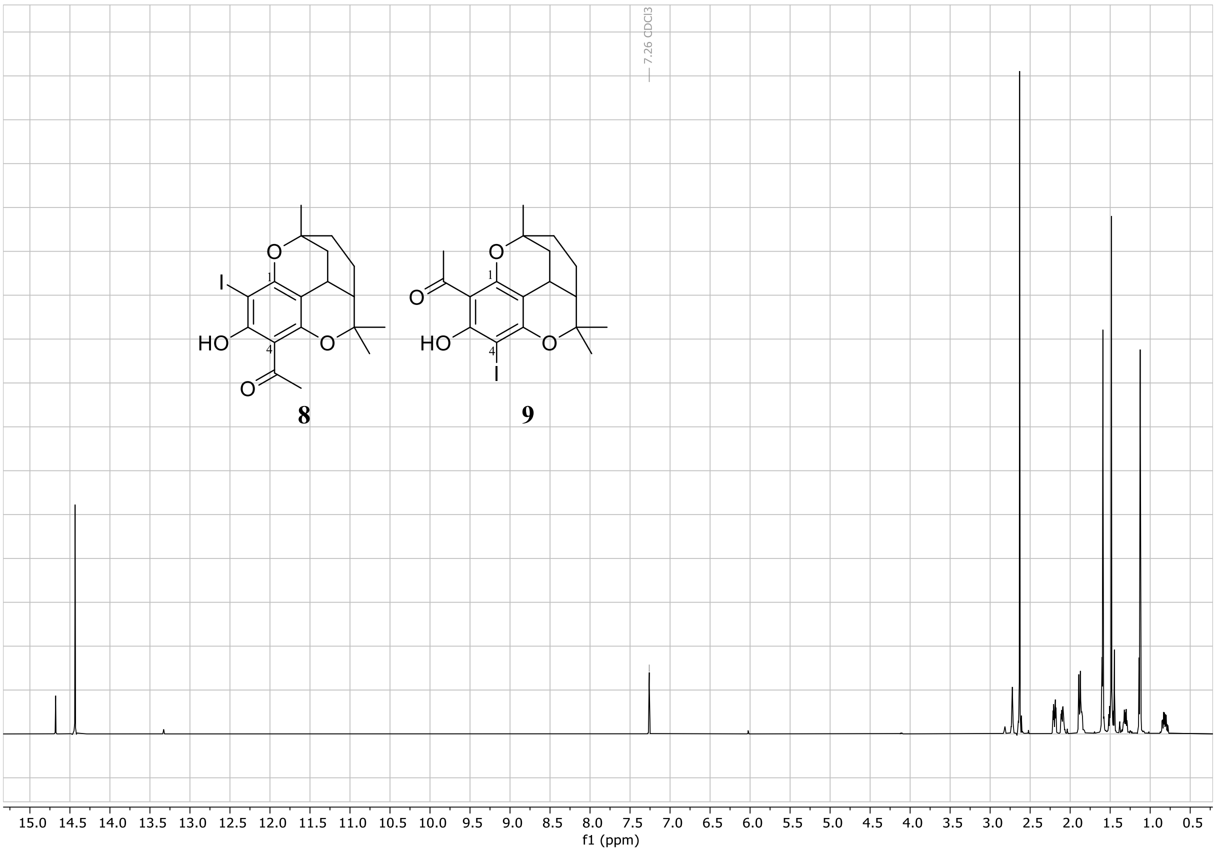

Figure S17. ^{1}H NMR (600 MHz, $CDCl_3$) spectrum of compound **8+9.**

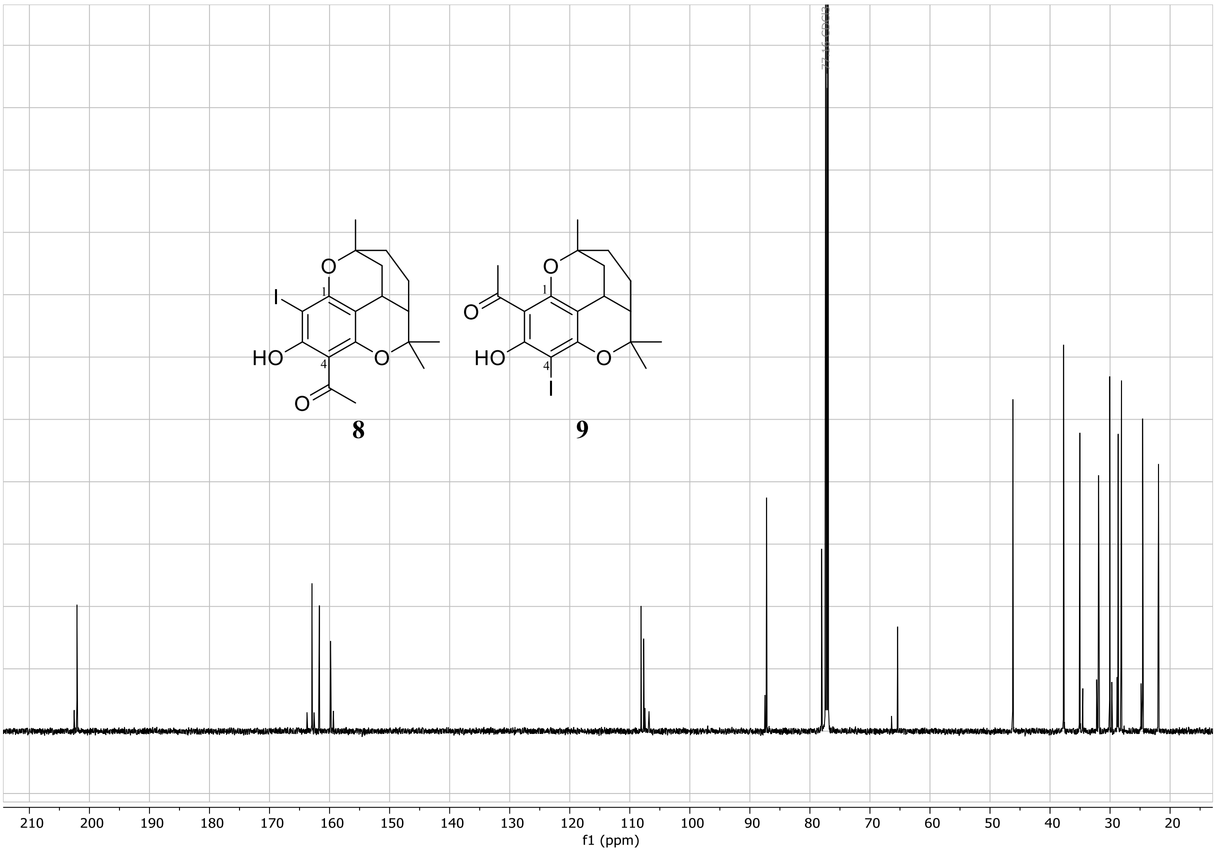

Figure S18. ^{13}C NMR (150 MHz, $CDCl_3$) spectrum of compound **8+9.**

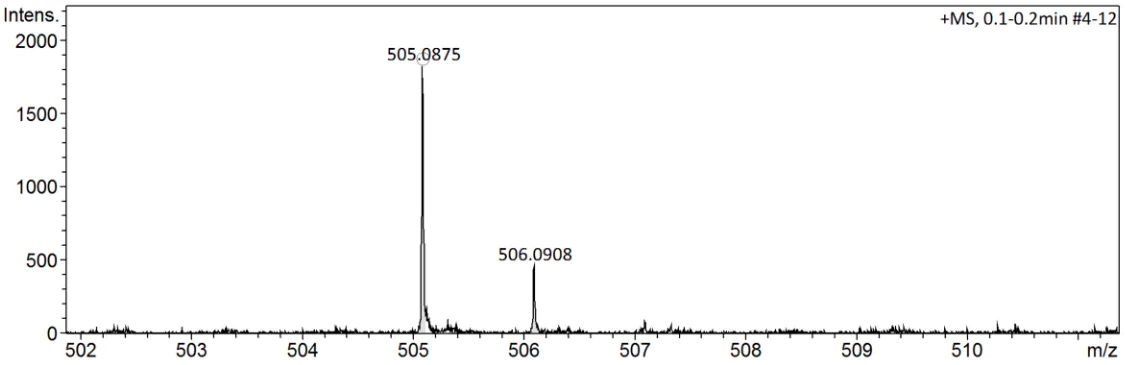

Meas. m/z	#	Ion Formula	m/z	err [ppm]	mSigma	# mSigma	Score	rdb	e⁻ Conf	N-Rule
505.0875	1	C24H26IO4	505.0870	-0.9	25.3	1	87.70	11.5	even	ok
	2	C25H22IN4	505.0884	1.8	31.4	2	59.59	16.5	even	ok
	3	C22H9N12O4	505.0864	-2.1	33.6	3	52.17	24.5	even	ok
	4	C25H17N2O10	505.0878	0.6	34.2	4	100.00	18.5	even	ok
	5	C23H5N16	505.0878	0.6	42.4	5	60.48	29.5	even	ok

Figure S19. HRESIMS of compound **10**.

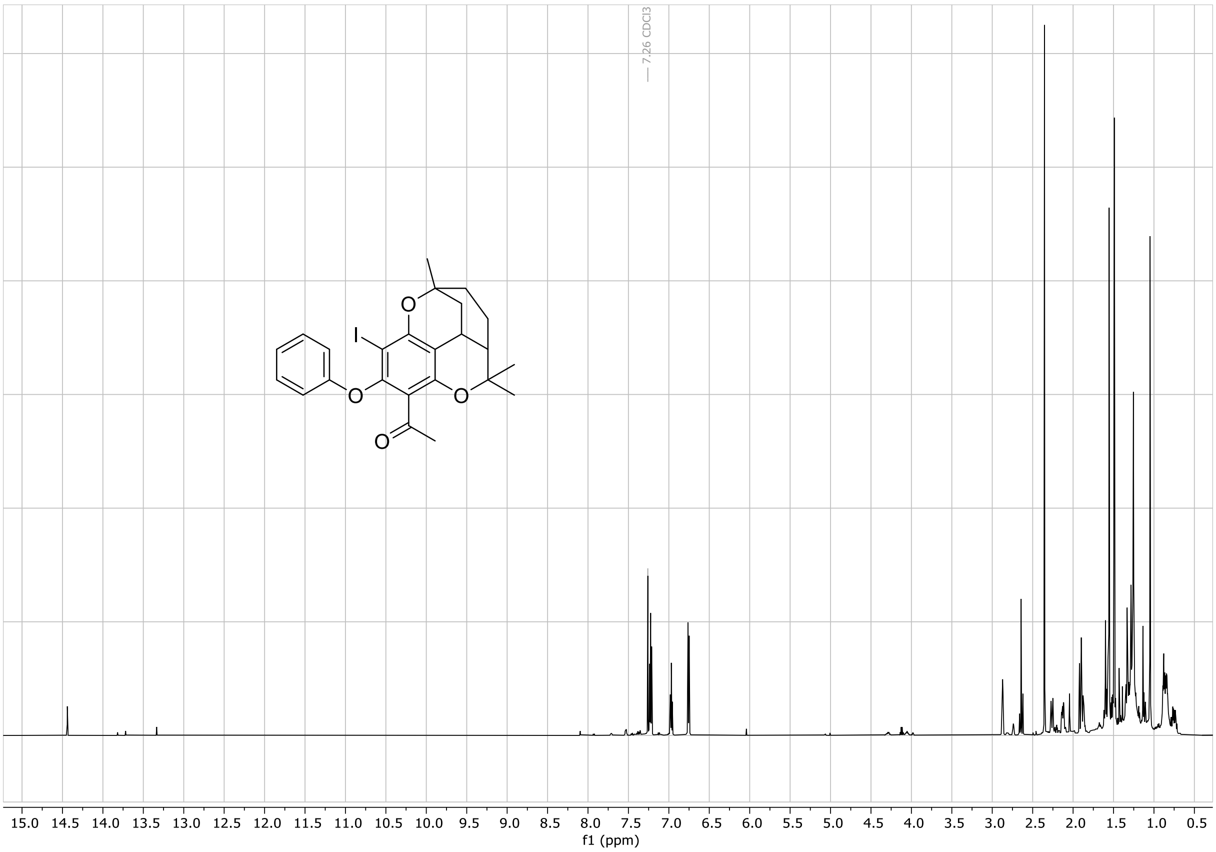

Figure S20. ^{1}H NMR (600 MHz, $CDCl_3$) spectrum of compound **10.**

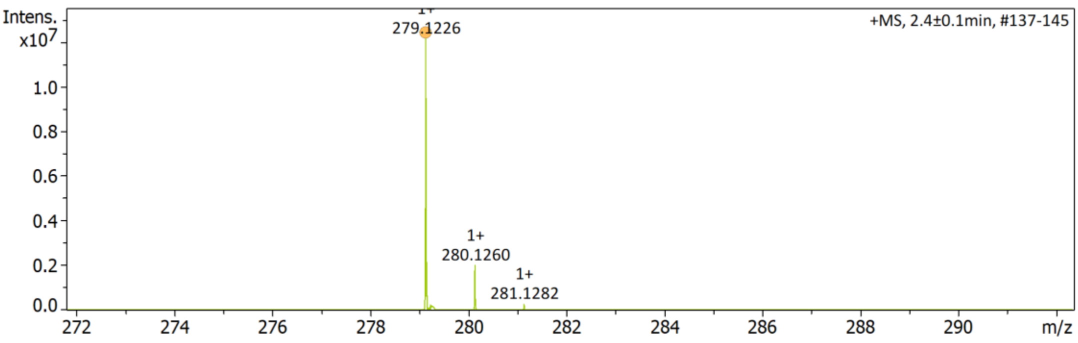

Meas. m/z	#	Ion Formula	m/z	err [ppm]	Mean err [ppm]	rdb (neutral)	N-Rule	e⁻ Conf	mSigma	Std I	Std Mean m/z	Std I VarNorm	Std m/z Diff	Std Comb Dev
279.1226	1	C15H19O5	279.1227	0.2	0.3	7.0	ok	even	1.3	2.8	n.a.	n.a.	n.a.	n.a.
279.1226	2	C16H15N4O	279.1240	5.0	4.6	12.0	ok	even	14.1	23.6	n.a.	n.a.	n.a.	n.a.

Figure S21. HRESIMS of compound **11**.

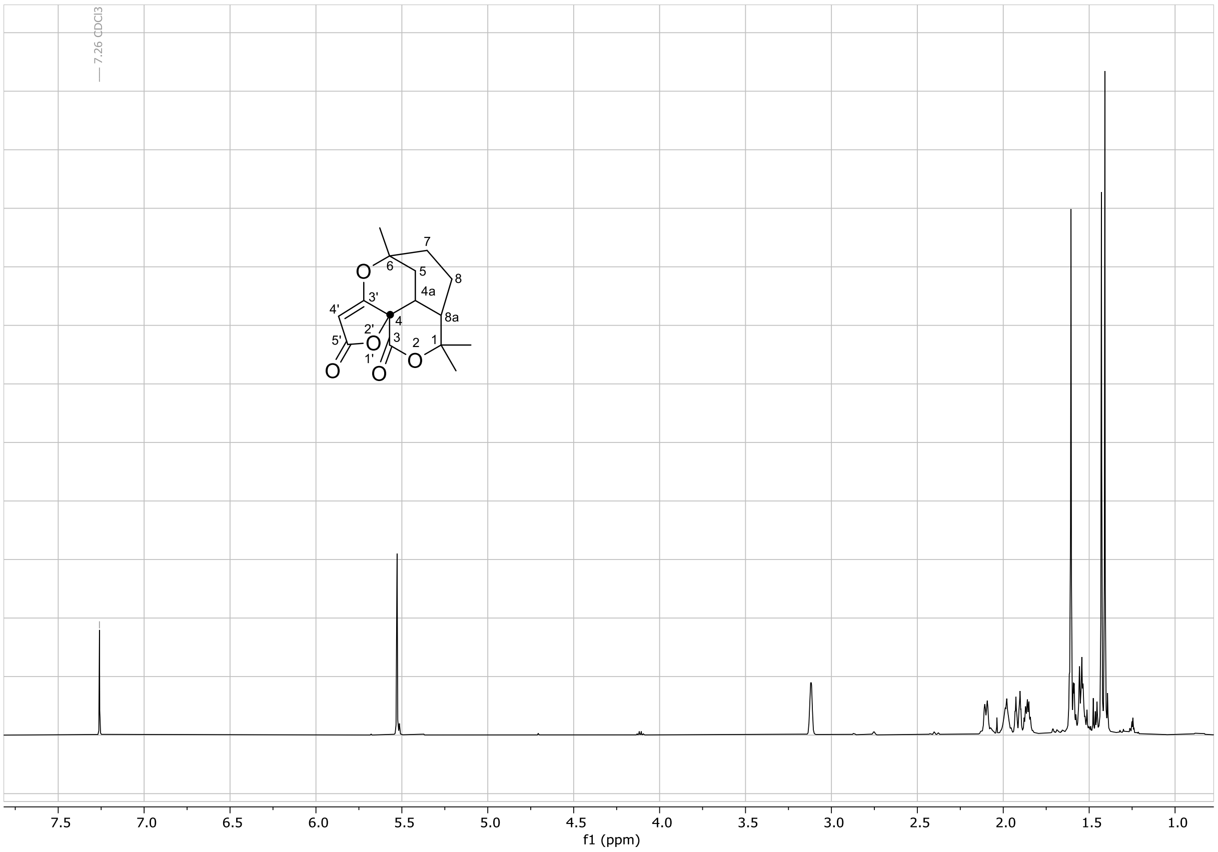

Figure S22. ^{1}H NMR (600 MHz, $CDCl_3$) spectrum of compound **11.**

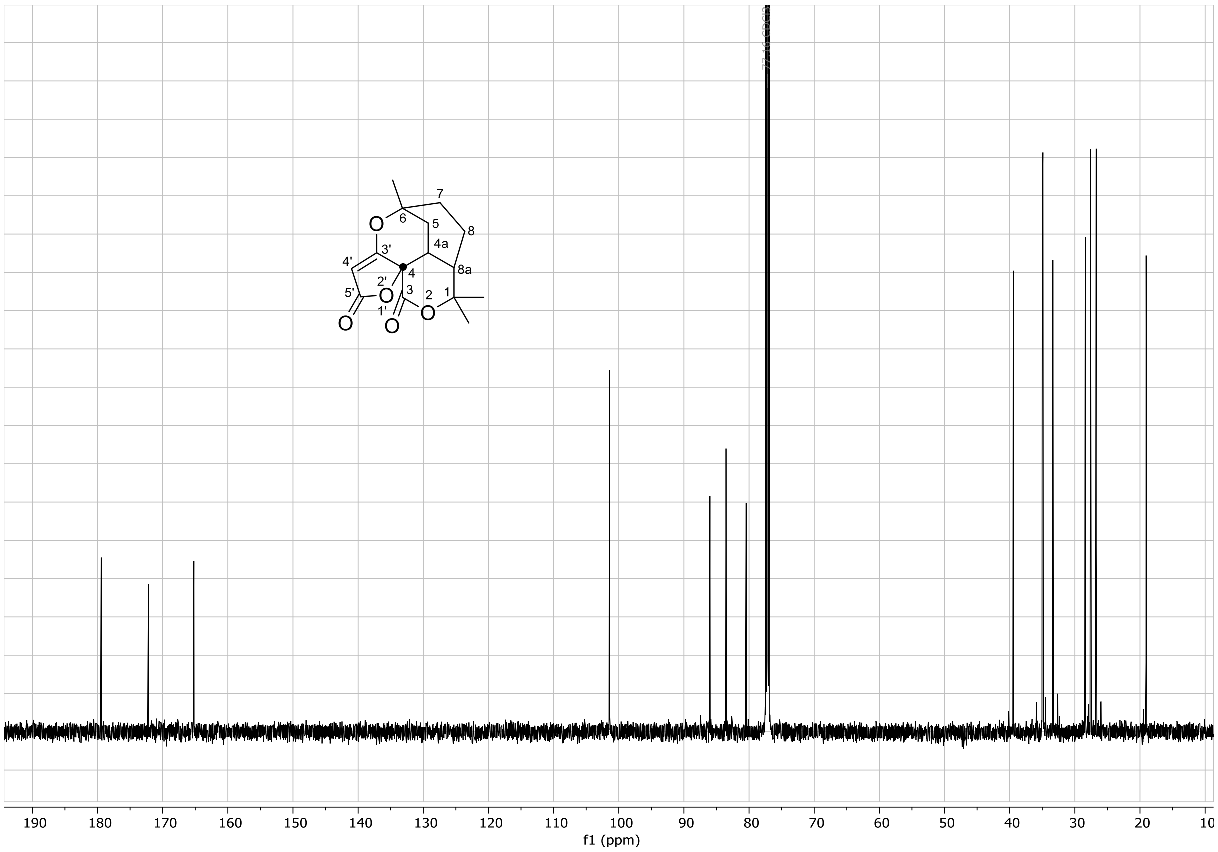

Figure S23. ^{13}C NMR (150 MHz, $CDCl_3$) spectrum of compound **11.**

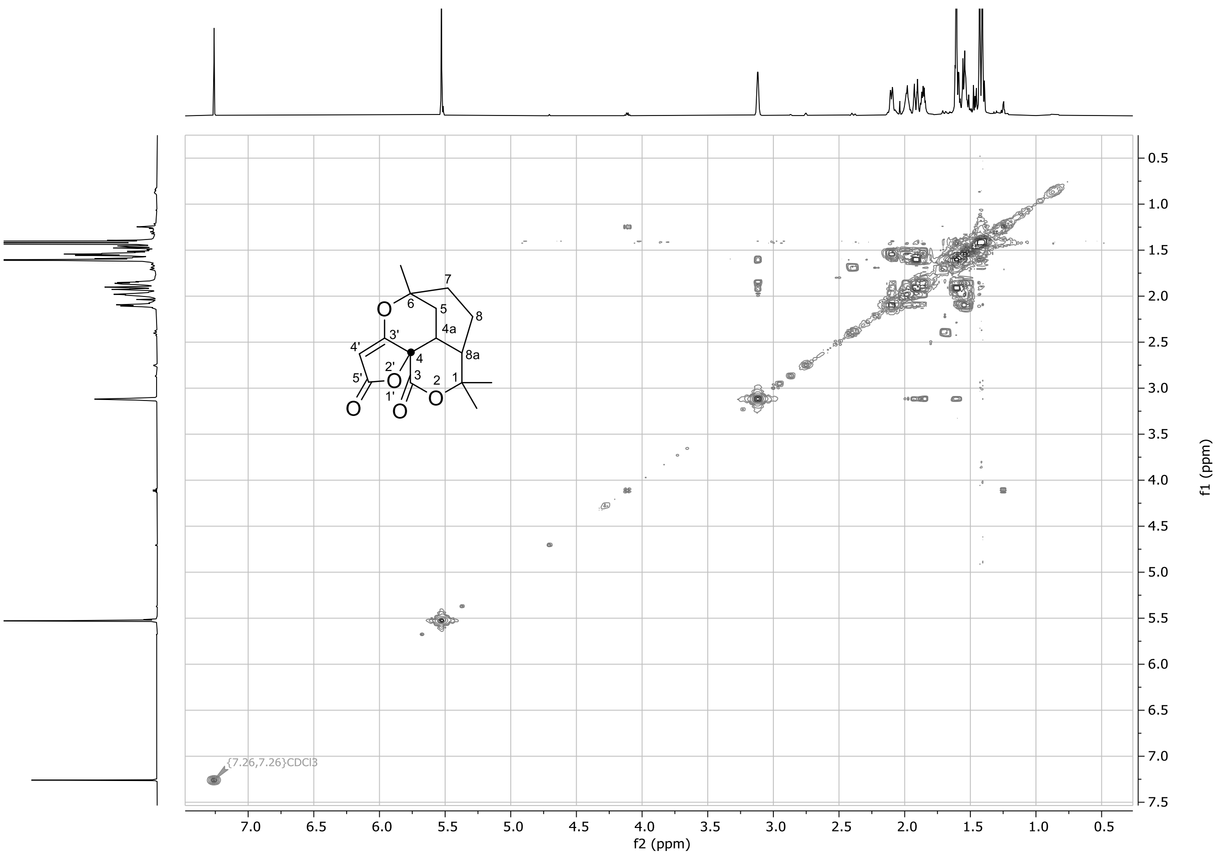

Figure S24. 2D-COSY-spectrum of compound **11.**

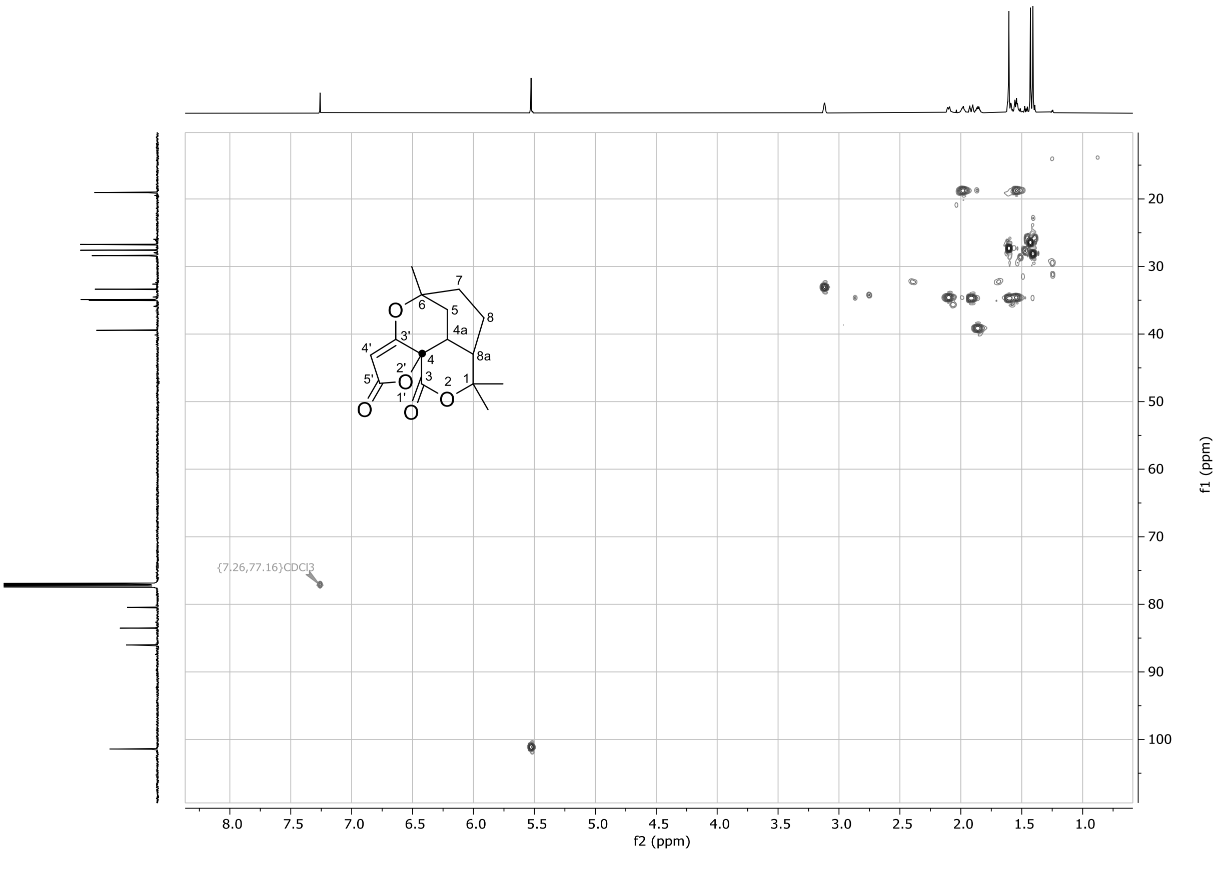

Figure S25. 2D-HSQC-spectrum of compound **11.**

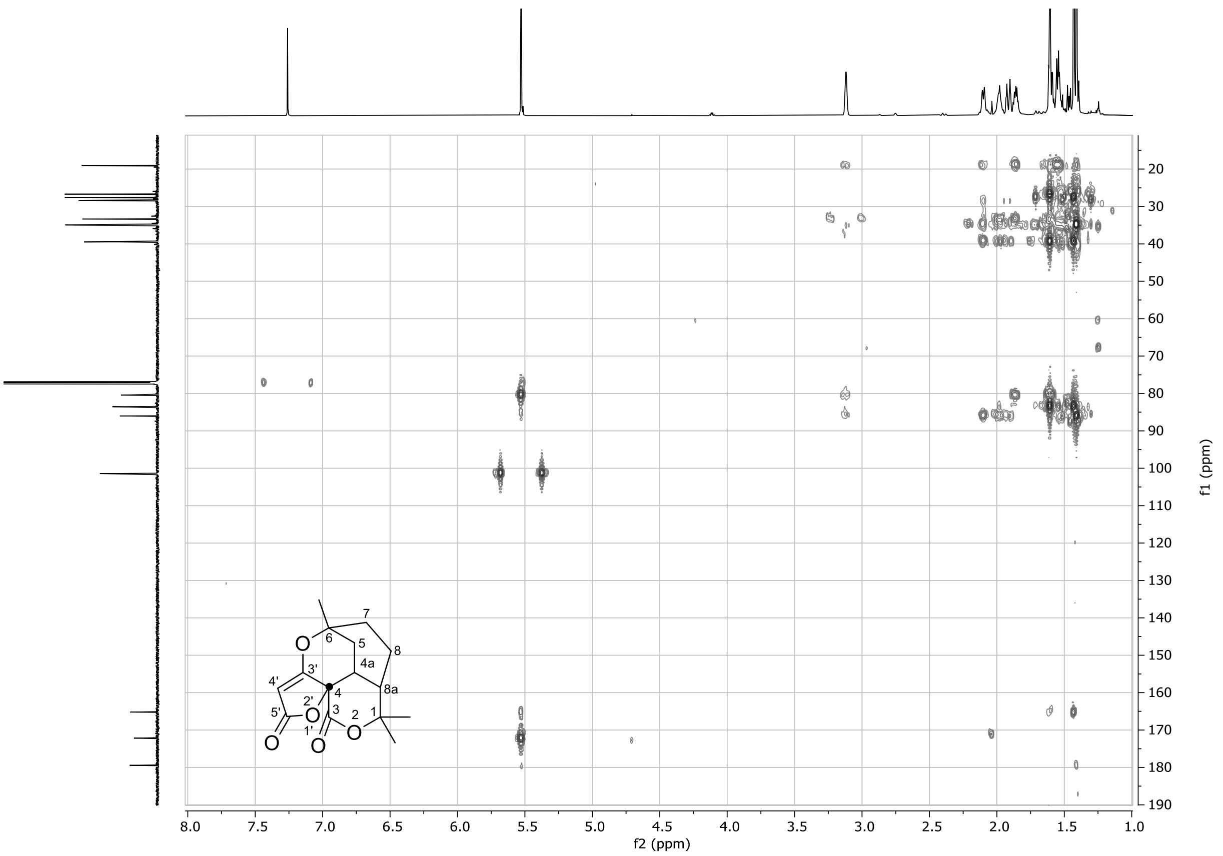

Figure S26. 2D-HMBC-spectrum of compound **11.**
